# A Feedback Loop Driven by H4K12 Lactylation and HDAC3 in Macrophages Regulates Lactate‐Induced Collagen Synthesis in Fibroblasts Via the TGF‐β Signaling

**DOI:** 10.1002/advs.202411408

**Published:** 2025-02-13

**Authors:** Ying Zou, Mibu Cao, Meiling Tai, Haoxian Zhou, Li Tao, Shu Wu, Kaiye Yang, Youliang Zhang, Yuanlong Ge, Hao Wang, Shengkang Luo, Zhenyu Ju

**Affiliations:** ^1^ Key Laboratory of Regenerative Medicine of Ministry of Education Institute of Aging and Regenerative Medicine College of Life Science and Technology Jinan University Guangzhou 510632 China; ^2^ Department of Plastic and Reconstructive Surgery Guangdong Second Provincial General Hospital Jinan University Guangzhou 510403 China; ^3^ R&D Center Infinitus (China) Company Ltd Guangzhou 510640 China; ^4^ Department of Cardiology Guangdong Provincial Cardiovascular Institute Guangdong Provincial People's Hospital Guangdong Academy of Medical Sciences Guangzhou 510080 China; ^5^ Department of Anesthesiology The First Affiliated Hospital Jinan University Guangzhou 510632 China

**Keywords:** collagen synthesis, fibroblasts, H4K12 lactylation, lactate, macrophages

## Abstract

The decrease in fibroblast collagen is a primary contributor to skin aging. Lactate can participate in collagen synthesis through lysine lactylation by regulating gene transcription. However, the precise mechanism by which lactate influences collagen synthesis requires further investigation. This study demonstrates that the depletion of macrophages mitigates the stimulating effect of lactate on collagen synthesis in fibroblasts. Through joint CUT&Tag and RNA‐sequencing analyses, a feedback loop between H4K12 lactylation (H4K12la) and histone deacetylase 3 (HDAC3) in macrophages that drives lactate‐induced collagen synthesis are identified. Macrophages can uptake extracellular lactate via monocarboxylate transporter‐1 (MCT1), leading to an up‐regulation of H4K12la levels through a KAT5‐KAT8‐dependent mechanism in response to Poly‐L‐Lactic Acid (PLLA) stimulation, a source of low concentration and persistent lactate, thereby promoting collagen synthesis in fibroblasts. Furthermore, H4K12la is enriched at the promoters of TGF‐β1 and TGF‐β3, enhancing their transcription. Hyperlactylation of H4K12la inhibits the expression of the eraser HDAC3, while the activation of HDAC3 reduces H4K12la in macrophages and suppresses collagen synthesis in fibroblasts. In conclusion, this study illustrates that macrophages play a critical role in lactate‐induced collagen synthesis in the skin, and targeting the lactate‐H4K12la‐HDAC3‐TGF‐β axis may represent a novel approach for enhancing collagen production to combat skin aging.

## Graphical abstract

Low concentrations of lactate are transported to macrophages via MCT1, which upregulates the levels of histone H4K12la and promotes the transcription and secretion of TGF‐β in macrophages. TGF‐β subsequently enters fibroblasts through TGF‐β receptors, stimulating collagen synthesis. Thus, lactate serves as a significant target for enhancing endogenous collagen synthesis and facilitating skin rejuvenation.

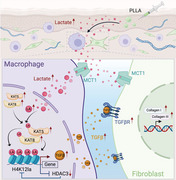

## 1 Introduction

Skin aging is a complex physiological process, primarily characterized by a reduction in fibroblast collagen, which is a significant contributor to skin laxity and wrinkle formation.^[^ ^1^ ^]^ As individuals age, the synthesis of fibroblast collagen diminishes while its degradation escalates, leading to a net decrease in total collagen and subsequently resulting in skin aging.^[^ ^2^ ^]^ Skin aging can be categorized into endogenous aging, which is predominantly influenced by genetic factors and occurs naturally over time, and exogenous aging, which is driven by environmental influences such as ultraviolet radiation and pollution.^[^ ^3^ ^]^ Concurrently, the antioxidant system becomes compromised, and the levels of reactive oxygen species (ROS) rise, further exacerbating collagen damage.^[^ ^4^ ^]^

Lactate is a significant product of glycolysis, particularly when cells are deprived of oxygen or when energy demand increases. In such conditions, glucose is converted into pyruvate via the glycolytic pathway and subsequently reduced to lactate by lactate dehydrogenase.^[^ ^5^ ^]^ Lactate not only provides energy for cells under anaerobic conditions but also shuttles between cells through lactate transporters (MCTs) to maintain energy balance and support immune responses.^[^ ^6^ ^]^ Furthermore, recent discoveries have revealed that lactate acts as a multifunctional signaling molecule capable of regulating immune response,^[^ ^7^ ^]^ angiogenesis,^[^ ^8^ ^]^ and tissue regeneration.^[^ ^9^ ^]^ It was first identified by Zhang et al. as a signaling molecule that regulates gene transcription through a novel post‐transcriptional modification (PTM) of proteins known as “lactylation”.^[^ ^10^ ^]^ Zhang et al. reported that modulation of intracellular lactate production in macrophages affects histone lactylation (Kla) levels in a dose‐dependent manner.^[^ ^10^ ^]^ Additionally, recent studies have uncovered that lactate can promote non‐histone lactylation during immune responses and tissue regeneration. For instance, Yang et al. found that sepsis increases serum lactate levels, which in turn promotes HMGB1 lactylation, further increasing endothelial cell permeability.^[^ ^11^ ^]^ These studies suggest that lactate may influence gene transcription via lactylation and is directly or indirectly involved in various pathological and physiological conditions.

In recent years, lactate has garnered increasing attention as a factor that stimulates endogenous collagen synthesis.^[^ ^12^ ^]^ Our recent study reported that the supply of Poly‐L‐Lactic Acid (PLLA), a source of low concentration and slow‐release lactate, promotes collagen synthesis in fibroblasts by inducing non‐histone protein LTBP1 lactylation at lysine 752. This finding provides valuable insights into the role of lactylation modification in skin rejuvenation.^[^ ^13^ ^]^ A high concentration of lactate is detrimental to skin collagen synthesis. Elevated lactate levels in organisms, particularly within inflammatory and disease contexts, can exacerbate cellular inflammatory reactions, this effect is especially pronounced in tumor microenvironments and immune‐mediated inflammatory diseases.^[^ ^14^, ^15^, ^16^ ^]^ Consequently, we have opted to use Poly‐L‐lactic acid (PLLA) to deliver low concentrations of lactate to the skin over an extended period, rather than administering lactate directly, a method validated in our previous study.^[^ ^13^ ^]^

Following the injection of PLLA into the skin, lactate is gradually released, leading to the recruitment of macrophages. Both macrophages and fibroblasts are involved in the collagen synthesis stimulated by lactate. As critical immune cells, macrophages play a significant role in tissue regeneration; they are primarily responsible for eliminating bacteria, viruses, and other harmful substances while also regulating inflammation, promoting wound healing, and facilitating the removal of pathogens.^[^ ^17^ ^]^ Upon skin injury or infection, macrophages migrate to the affected area, clear bacteria, and pathogens, and release inflammatory mediators to modulate the inflammatory response.^[^ ^18^ ^]^ Additionally, macrophages secrete growth factors and cytokines that enhance the proliferation and migration of skin fibroblasts. They also participate in the regulation of collagen synthesis and degradation, thereby aiding in skin regeneration.^[^ ^19^ ^]^ Although our recent article reports that lactate promotes collagen synthesis through non‐histone protein lactylation.^[^ ^13^ ^]^ The specific role of macrophages in this process warrants further investigation.

The purpose of this paper is to explore the role of macrophages in lactate‐induced collagen synthesis. In the current study, we found that the depletion of macrophages via clodronate‐liposomes attenuates lactate‐induced collagen synthesis in fibroblasts, indicating the indispensable role of macrophages in combating skin aging. Mechanically, lactate enters macrophages through MCT1, and the lactylation writers KAT5 and KAT8 cooperatively promote the lactylation of histone H4 at lysine residue 12 (H4K12la) in lactate‐stimulated macrophages. Additionally, CUT&Tag analysis revealed that H4K12la enhances the transcription of TGF‐β1 and TGF‐β3, these genes are closely associated with collagen production in fibroblasts. Furthermore, we identified a feedback loop between H4K12la and the lactylation eraser HDAC3 in macrophages that regulates collagen synthesis in the skin. Collectively, our data establish a connection between macrophage‐fibroblast crosstalk in lactate‐derived lactylation and suggest the presence of a feedback loop between H4K12la and HDAC3 in skin collagen synthesis, highlighting a promising target for anti‐skin aging interventions.

## 2 Results

### 2.1 PLLA is Safe for Macrophages and Promotes their Migration and Phagocytosis

**Figure 1:**
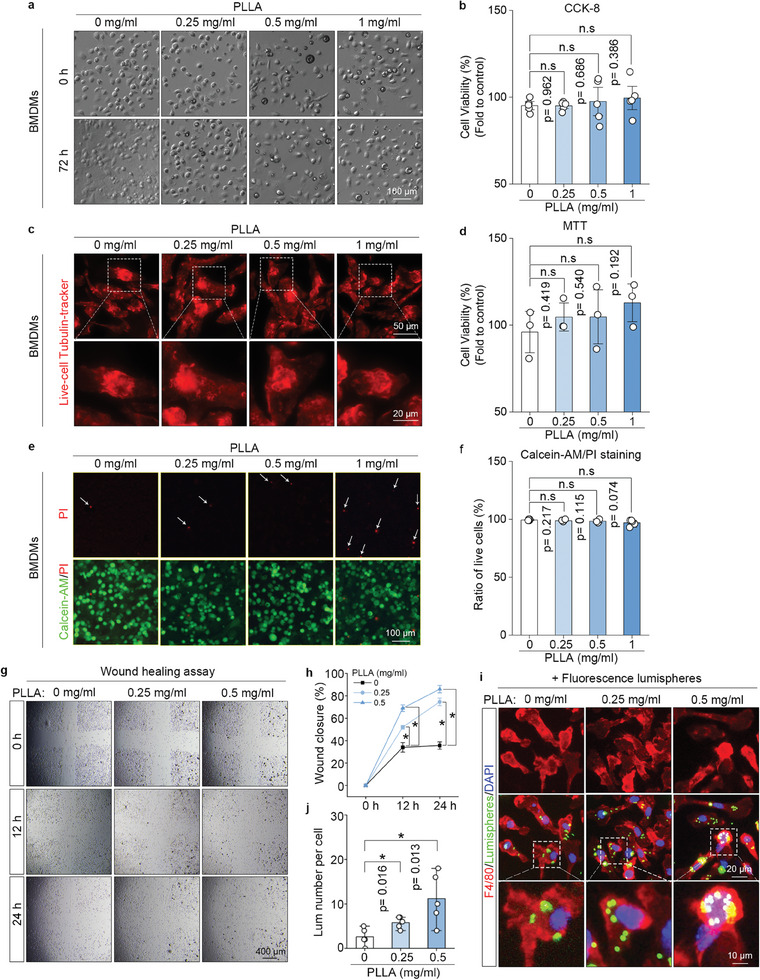
PLLA is safe to macrophages and promotes their migration and phagocytosis. a) BMDMs were treated with varying concentrations (0, 0.25, 0.5, and 1 mg mL^−1^) of PLLA for 72 h. Cellular morphology was observed using a phase contrast microscope. b) Cell viability was evaluated using the Cell Counting Kit‐8 (CCK8). “n.s” indicating no significance. c) BMDMs were treated with (0, 0.25, 0.5, and 1 mg mL^−1^) of PLLA for a duration of 72 h. The live cell marker, tubulin, was stained using a Live‐cell Tubulin‐tracker. d) Cell viability was measured using the MTT assay. “n.s” indicating no significance. e,f) Calcein‐AM/PI staining was performed to measure the percentages of live cells, “n.s” indicating no significance. g,h) The effects of PLLA in macrophages migration were examined by wound healing assay, representative images of the wound healing assay in BMDMs and statistical analysis of wound healing assay in BMDMs. ^*^ *p* < 0.05. i,j) Double immunofluorescence staining for F4/80‐stained macrophages (red fluorescence) and fluorescence lumispheres (green fluorescence), and quantification of the lumispheres number per macrophage. ^*^ *p* < 0.05.

The safety of PLLA on macrophages, specifically the RAW264.7 cells and primary bone marrow‐derived macrophages (BMDMs), was evaluated 72 h after treatment with increasing concentrations of PLLA (0, 0.25, 0.5, and 1 mg mL^−1^). Cell viability and survival were assessed using the CCK‐8 assay and Calcein‐AM/PI staining, respectively. The CCK‐8 assay demonstrated that cell viability did not change following PLLA treatment in both RAW264.7 (Figure S1a,b, Supporting Information) and BMDMs (**Figure** **1**a,b). In live cell labeling experiments, α‐tubulin served as a model protein, and live cell tubulin‐tracker was employed to label both control BMDMs and PLLA‐treated BMDMs (Figure 1c). The MTT assay demonstrated that cell viability remained unchanged following PLLA treatment in BMDMs (Figure 1d). Calcein‐AM/PI staining indicated no significant increase in dead cells after PLLA treatment in both RAW264.7 (Figure S1c,d, Supporting Information) and BMDMs (Figure 1e,f). These data suggest that low concentrations of PLLA, as a source of lactate, are safe for macrophages. Subsequently, the effects of PLLA on the migration and phagocytosis of macrophages were investigated. The wound healing assay showed a significant induction of migration in BMDMs at 12 and 24 h post‐PLLA treatment at concentrations of 0.25 and 0.5 mg mL^−1^ (Figure 1g,h). Next, we assessed whether lactate influences the phagocytosis of macrophages. Fluorescent lumispheres were added to either control or PLLA‐treated BMDMs for 4 h. Immunofluorescence staining for F4/80 (a macrophage marker) illustrated that the number of phagocytosed lumispheres per cell in PLLA‐treated BMDMs was significantly greater than that in the control group, in a dose‐dependent manner (Figure 1i,j). These findings indicate that lactate released from PLLA enhances the migration and phagocytosis abilities of macrophages in a dose‐dependent manner.

### 2.2 Macrophages Promote Lactate‐Induced Collagen Synthesis in Fibroblasts Both In Vivo and In Vitro

**Figure 2:**
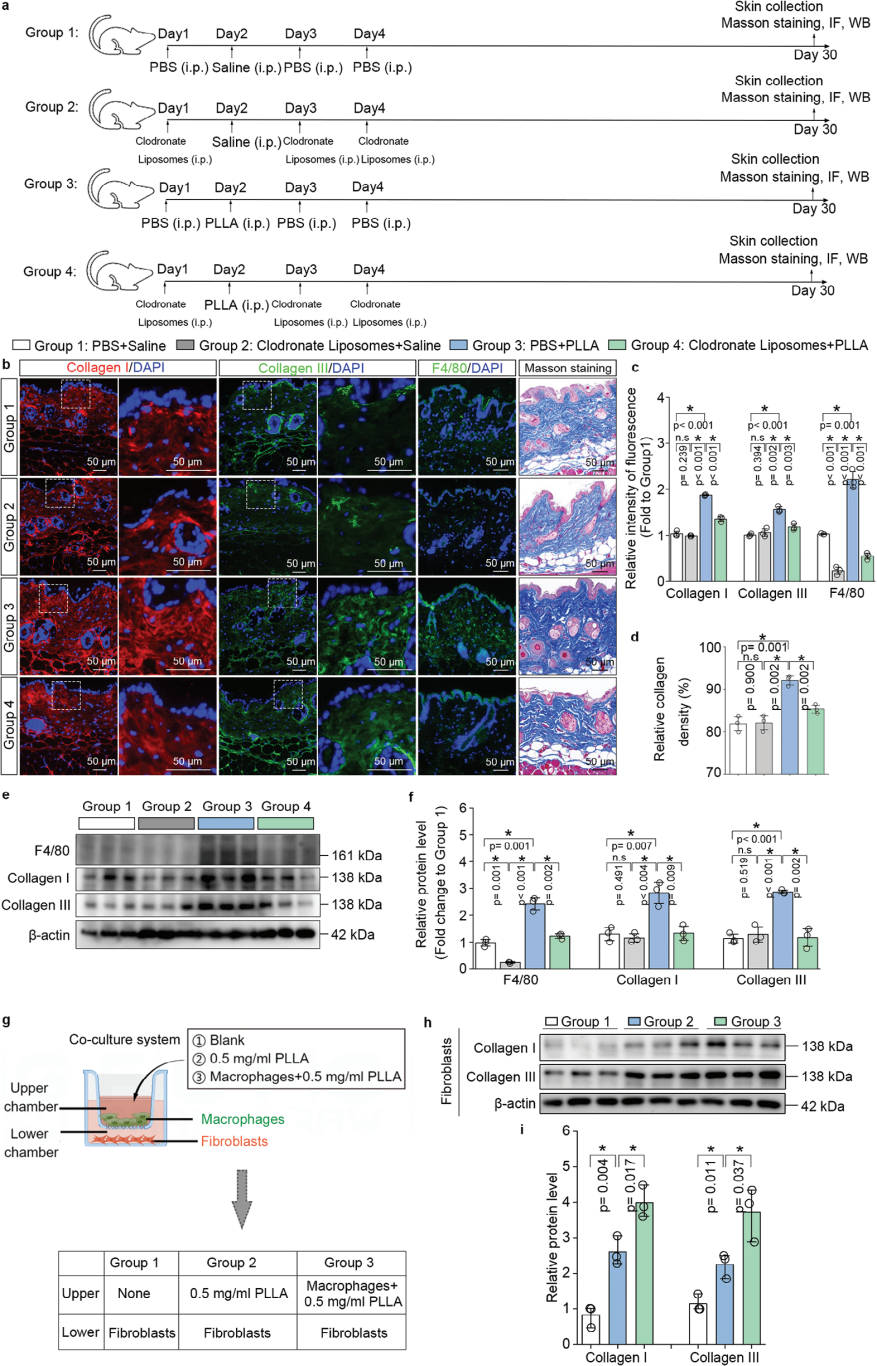
Macrophages promote lactate‐induced collagen synthesis in fibroblasts both in vivo and in vitro. a) Experimental protocol for the animal procedure including injections, sampling, and experiment termination for histological studies and western blotting. Group 1 was termed as control group; Group 2 was injected with clodronate liposomes only; Group 3 was injected with PLLA only; Group 4 was combination of PLLA and clodronate liposomes injection. PBS and saline were used for solvent controls of clodronate liposomes and PLLA, respectively. b) Collagen I (red fluorescence) and collagen III (green fluorescence) immunofluorescence in skin, F4/80 (green fluorescence) immunofluorescence in skin, and blue fluorescence refers to DAPI. And histological analysis of Masson staining in each group. c) Relative density of fluorescence density was normalized to group 1. ^*^ *p* < 0.05. d) The relative collagen density was quantified, refers to Masson staining. ^*^ *p *< 0.05. e,f) Western blotting analysis of proteins from skin samples, the relative protein levels of F4/80, collagen I, and collagen III were quantified. ^*^ *p* < 0.05. g) Macrophage‐fibroblast co‐culture in vitro system. Macrophages were seeded in the upper chamber; fibroblasts were seeded in the lower chamber. h,i) Western blotting analysis of collagen I and collagen III protein levels in fibroblast (lower chamber), the relative protein levels of collagen I and collagen III were quantified. ^*^ *p* < 0.05.

We investigated the role of macrophages in lactate‐induced collagen synthesis through in vivo experiments using mice, where we employed clodronate liposomes to deplete macrophages following the injection of PLLA. Skin samples were collected on day 30 for examination of collagen production via immunofluorescence staining, Masson staining, and western blotting (**Figure** **2**a). Our analysis focused on whether macrophages contribute to lactate‐induced collagen synthesis in the skin. In comparison to normal skin, the density of macrophages was significantly reduced in the skin of mice injected with clodronate liposomes alone; however, the levels of collagen I and collagen III remained unchanged (Figure 2b,c). We observed that the protein levels of collagen I and collagen III were significantly up‐regulated after PLLA injection. Notably, our findings revealed that collagen production (Figure 2b,c) and collagen density (Figure 2b,d) in the skin were significantly heightened in response to lactate stimulation, however, this phenomenon was reversed by macrophage depletion. To further substantiate this hypothesis, we performed western blotting for F4/80, collagen I, and collagen III examination (Figure 2e,f). Subsequently, we established a macrophage‐fibroblast co‐culture system in vitro (Figure 2g). BMDMs were seeded in the upper chamber with or without PLLA addition, allowing direct access to PLLA‐released lactate and cytokines secreted by macrophages into the lower chamber (Figure 2g). Interestingly, the production of collagen I and collagen III were significantly increased in the presence of macrophages following PLLA addition (Figure 2h,i). Overall, these results suggest that macrophages play a vital role in lactate‐induced collagen synthesis in fibroblasts.

### 2.3 Macrophages Uptake Lactate Via MCT1 and H4K12 Lactylation (H4K12la) is Increased in Response to Lactate Stimulation

**Figure 3:**
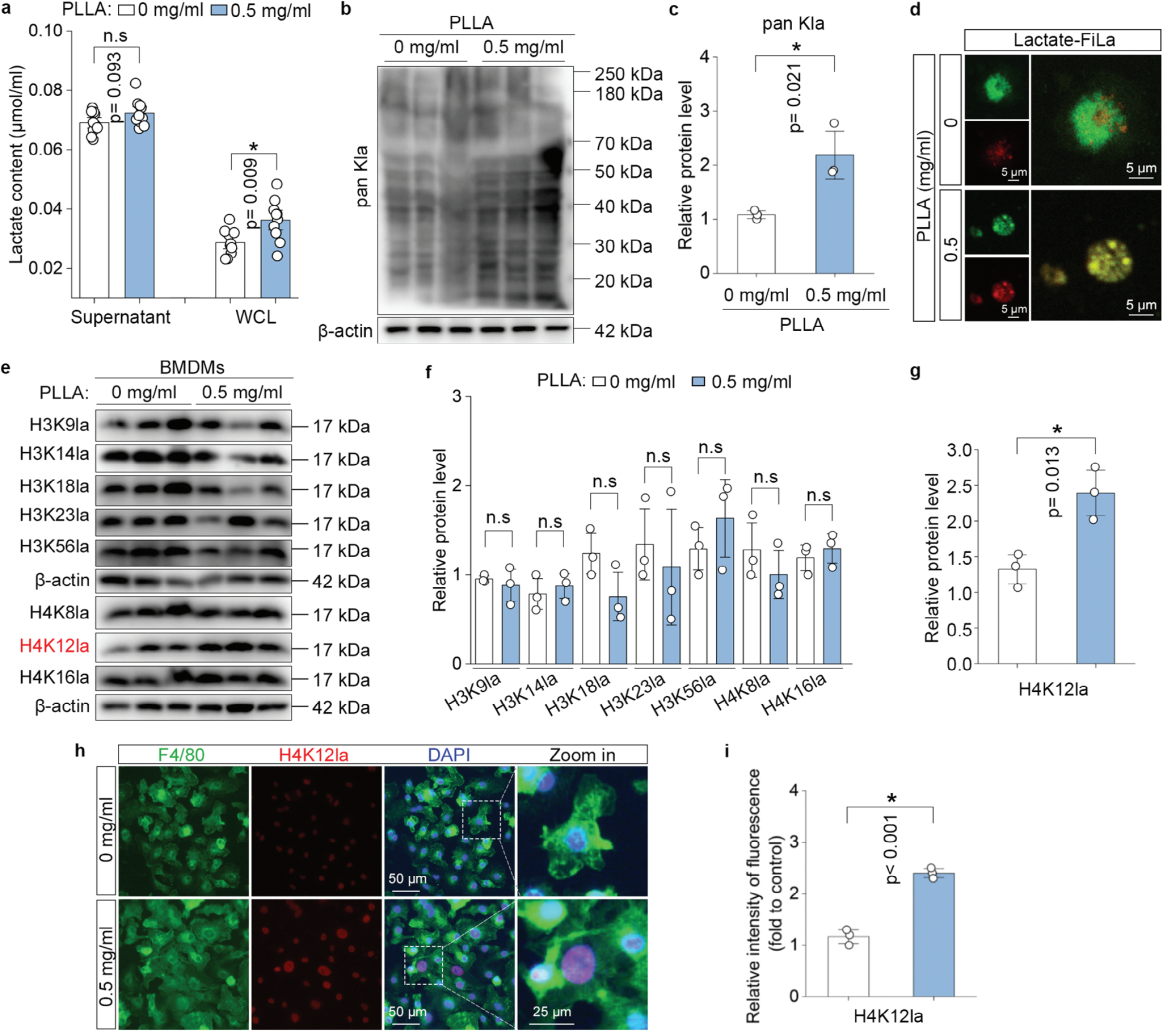
H4K12 lactylation (H4K12la) is increased in response to lactate stimulation in macrophages. a) BMDMs were treated with or not treated with 0.5 mg mL^−1^ PLLA for 72 h, lactate contents in the cell free supernatant and whole cell lysate from PLLA‐treated BMDMs and control BMDMs. “n.s” indicating no significance, ^*^ *p* < 0.05. b,c) Western blotting analysis of pan Kla levels in BMDMs treated or not treated with PLLA, and the relative pan Kla levels were normalized to β‐actin. ^*^ *p *< 0.05. d) Fluorescence images of macrophages treated with 0.5 mg mL^−1^ PLLA for 72 h using FiLa sensor to visualize intracellular lactate. e–g) Western blotting analysis of H3K9la, H3K14la, H3K18la, H3K23la, H3K56la, H4K8la, H4K12la, and H4K16la in BMDMs treated or not treated with PLLA, and the relative the indicated histone lactylation levels were normalized to β‐actin. “n.s” indicating no significance, ^*^ *p* < 0.05. h,i) Double immunofluorescence staining for F4/80‐stained macrophages (green fluorescence) and H4K12la (red fluorescence), and quantification of H4K12la fluorescence normalized to control group. ^*^ *p* < 0.05.

**Figure 4:**
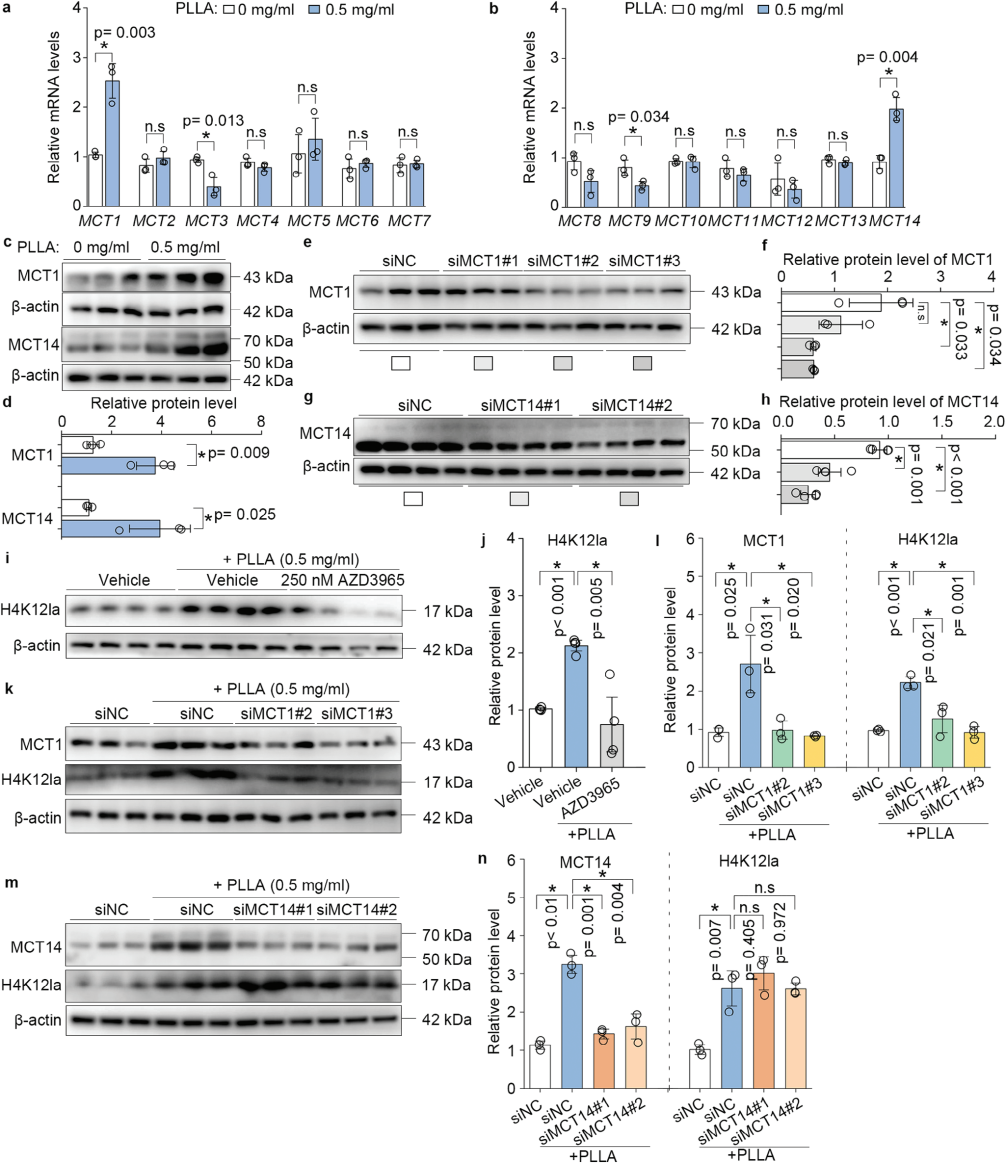
Macrophages uptake lactate via MCT1 after being treated with PLLA. a,b) qPCR analysis of MCT1‐MCT14 in BMDMs, the mRNA levels were normalized to β‐actin. “n.s” indicating no significance, ^*^ *p* < 0.05. c,d) Western blotting analysis of MCT1 and MCT14 in BMDMs treated or not treated with PLLA, and the relative MCT1 and MCT14 levels were normalized to β‐actin. ^*^ *p* < 0.05. e,f) Three pairs of MCT1 siRNAs were designed and synthesized, and the silencing efficiency of these siRNAs in macrophages was confirmed by western blotting analysis of MCT1 expression, the relative MCT1 levels were normalized to β‐actin. “n.s” indicating no significance, ^*^ *p* < 0.05. g,h) Two pairs of MCT14 siRNAs were designed and synthesized, and the silencing efficiency of these siRNAs in macrophages was confirmed by western blotting analysis of MCT14 expression, with relative levels normalized to β‐actin. ^*^ *p* < 0.05. i,j) MCT1 inhibitor AZD3965 or k,l) MCT1 siRNAs were used for inhibiting MCT1 in macrophages in the presence of PLLA, western blotting analysis of MCT1 and H4K12la in BMDMs and the relative MCT1 and H4K12la levels were normalized to normalized to β‐actin. ^*^ *p* < 0.05. m,n) MCT14 siRNAs were utilized to inhibit MCT14 in macrophages in the presence of PLLA. Western blotting analysis was conducted to assess H4K12la levels in BMDMs, with the relative levels of MCT14 and H4K12la normalized to β‐actin. “n.s” indicating no significance, ^*^ *p* < 0.05.

Since lactate is continuously and slowly released from PLLA, we examined lactate content in the cell‐free supernatant and whole cell lysate (WCL) in BMDMs after PLLA treatment. Our findings indicated that lactate content was significantly increased in WCL of PLLA‐treated BMDMs (**Figure** **3**a). As previously reported, lactate serves as a substrate for lactylation,^[^ ^10^ ^]^ we found that PLLA caused a notable increase pan Kla in macrophages (Figure 3b,c). Furthermore, lactate levels were visualized using the FiLa sensor,^[^ ^20^ ^]^ revealing that intercellular lactate levels in PLLA‐treated BMDMs were significantly higher than in control cells (Figure 3d). We next investigated whether the up‐regulation of lactate content promoted histone lactylation in macrophages. We analyzed the lactylation levels of several well‐characterized lactylated residues in histones, including H3K9la, H3K14la, H3K18la, H3K23la, H3K56la, H4K8la, H4K12la, and H4K16la. Our observations indicated that the lactylation level of H4K12 was significantly up‐regulated in response to lactate stimulation (Figure 3e–g), and H4K12la levels were confirmed through immunofluorescence staining (Figure 3h,i). Given that monocarboxylate transporters (MCTs) have been previously reported to be responsible for lactate transport into the cell.^[^ ^21^ ^]^ First, qPCR was employed to assess the transcript levels of MCT1 through MCT14 in BMDMs treated with 0.5 mg mL^−1^ PLLA, the results suggested that only the mRNA levels of *MCT1* and *MCT14* were significantly elevated in PLLA‐treated BMDMs compared to the control cells (**Figure** **4**a,b). Then western blotting results also revealed that MCT1 and MCT14 levels were elevated in PLLA‐treated macrophages (Figure 4c,d). To confirm whether macrophages uptake lactate via MCT1 and MCT14, we established three pairs of MCT1 siRNA and two pairs of MCT14 siRNA. Western blotting results revealed that pairs #2 and #3 of MCT1 siRNA effectively silenced MCT1 expression, while pairs #1 and #2 of MCT14 siRNA effectively silenced MCT14 expression (Figure 4e–h). By combining this with a selective MCT1 inhibitor, AZD3965, we observed that MCT1 inhibition led to a decrease in H4K12 lactylation levels in the presence of PLLA (Figure 4i,j). Additionally, MCT1 silencing using siRNA significantly decreased the levels of H4K12 lactylation in the presence of PLLA (Figure 4k,l). However, silencing of MCT14 did not alter the increased levels of H4K12la in the presence of PLLA (Figure 4m,n). In conclusion, these results suggest that macrophages uptake lactate released from PLLA via MCT1, and H4K12 lactylation may represent an efficient mechanism to induce collagen synthesis in response to lactate stimulation

### 2.4 Lactylation Writers KAT5 and KAT8 Collaboratively Promotes H4K12la in Macrophages

**Figure 5:**
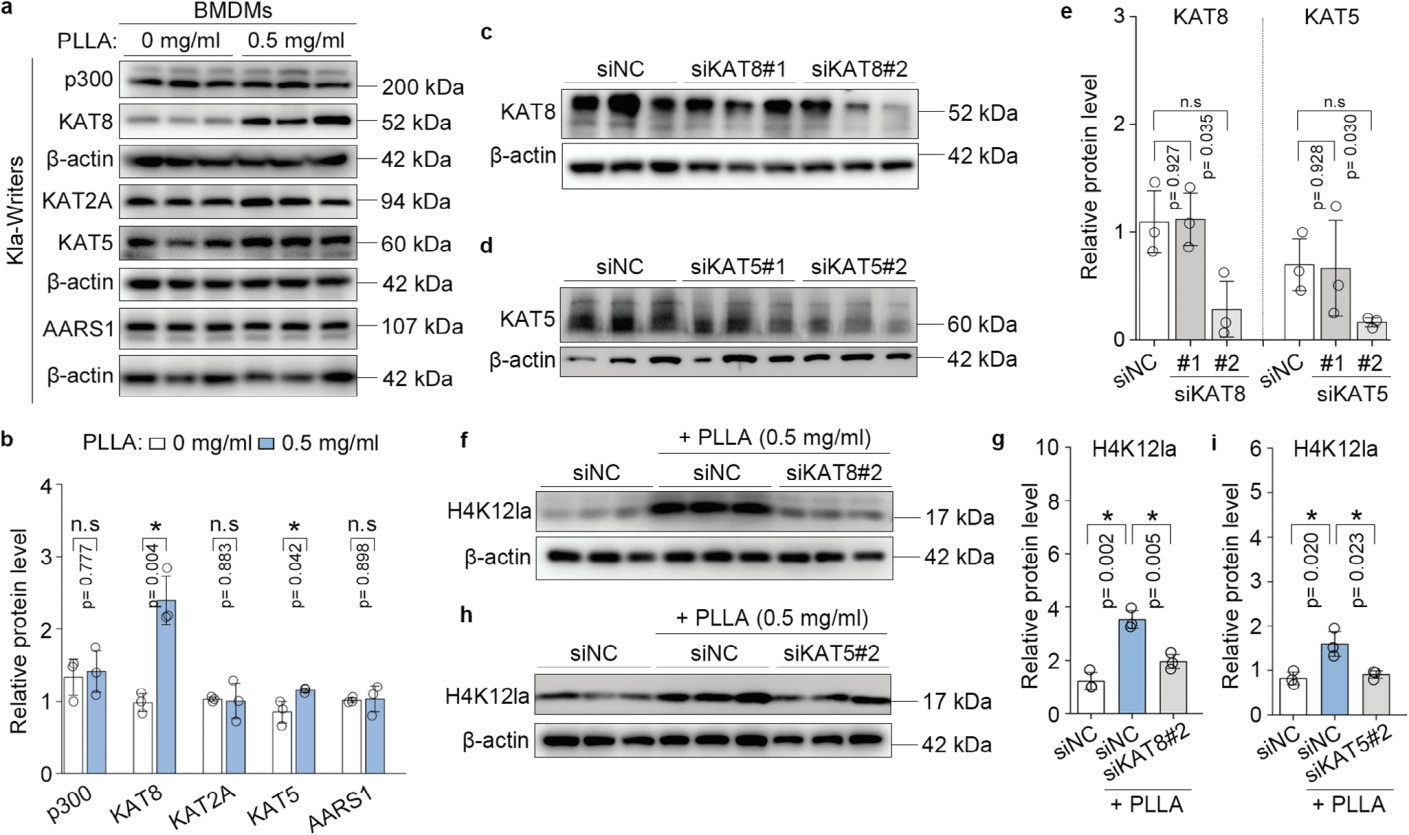
Lactylation writers KAT5 and KAT8 collaboratively promotes H4K12la in macrophages. a,b) Western blotting analysis of potential lactylation writers including p300, KAT8, KAT2A, KAT5, and AARS1 in BMDMs treated with or not treated with PLLA for 72 h, the relative protein levels were normalized to β‐actin. “n.s” indicating no significance, ^*^ *p* < 0.05. c–e) Western blotting analysis of KAT8, and KAT5 expression in siNC‐transfected, siKAT8‐transfected, siKAT5‐transfected BMDMs, the relative protein levels were normalized to β‐actin. “n.s” indicating no significance, ^*^ *p *< 0.05. f,g) Western blotting analysis of H4K12la levels in BMDMs after KAT8 knockdown in the presence of PLLA, the relative H4K12 levels were normalized to β‐actin. ^*^ *p *< 0.05. h,i) Western blotting analysis of H4K12la levels in BMDMs after KAT5 knockdown in the presence of PLLA, the relative H4K12 levels were normalized to β‐actin. ^*^ *p *< 0.05.

To identify the regulators of H4K12 lactylation, we examined the levels of five potential lactyl‐transferases (Kla writers), including p300, KAT8, KAT2A, KAT5, and AARS1 using western blotting. The results indicated that, compared to control cells, the protein levels of KAT5 and KAT8 were significantly increased in PLLA‐treated BMDMs (**Figure** **5**a,b). Furthermore, KAT8 and KAT5 siRNAs were established, and the efficiency of silencing KAT8 or KAT5 was confirmed (Figure 5c–e). To investigate whether KAT5 and KAT8 are responsible for transferring the lactyl group from lactyl‐CoA to histone H4K12, we tested the lactylation levels of H4K12 following KAT8 or KAT5 silencing. Notably, KAT8 knockdown abolished H4K12 lactylation in the presence of PLLA (Figure 5f,g). Similarly, KAT5 knockdown also eliminated H4K12 lactylation in the presence of PLLA (Figure 5h,i). Overall, KAT5 and KAT8 function as lactyl‐transferases (Kla writers) and collaboratively promote H4K12 lactylation in macrophages.

### 2.5 RNA‐seq Analysis Revealed ECM‐Receptor Interaction and TGF‐β Signaling are Involved in Macrophages in Response to Lactate Stimulation

**Figure 6:**
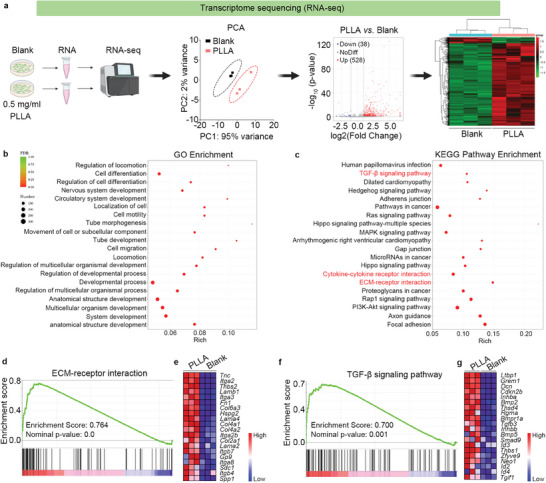
RNA‐seq analysis revealed ECM‐receptor interaction and TGF‐β signaling are involved in macrophages in response to lactate stimulation. a) BMDMs were exposed to 0.5 mg ml^−1^ PLLA for 72 h, while untreated cells served as the control group. RNA‐seq was performed on these cells, resulting in the identification of 528 up‐regulated genes and 38 down‐regulated genes in BMDMs. A heatmap was used to visualize the differential gene expression between the control group and PLLA‐treated‐BMDMs. b) Enriched GO analysis was conducted on the up‐regulated expressed genes. c) Analysis of KEGG pathway enrichment was conducted on the up‐regulated expressed genes, highlighting their involvement in critical processed related to ECM‐receptor interaction and TGF‐β signaling. d,e) GSEA analysis demonstrating ECM‐receptor interaction is positive correlation with PLLA treatment in BMDMs, a heatmap was used to visualize the top 20 up‐regulated genes involved in ECM‐receptor interaction. f,g) GSEA analysis demonstrating TGF‐β signaling pathway is positive correlation with PLLA treatment in BMDMs, a heatmap was used to visualize the top 20 up‐regulated genes involved in TGF‐β signaling.

To identify the potential genes in macrophages to regulate lactate‐induced collagen synthesis in fibroblasts. RNA‐sequencing was performed to identify differentially expressed genes after PLLA treatment in BMDMs. Subsequent analysis identified 528 up‐regulated genes and 38 down‐regulated genes (**Figure** **6**a). These up‐regulated genes were classified by gene ontology (GO) analysis into various functional categories, including “Developmental process”, “Anatomical structure development”, “Multicellular organism development”, and “System development” (Figure 6b). Additionally, KEGG analysis of these up‐regulated genes indicated enrichment in pathways related to the “TGF‐β signaling pathway”, “Cytokine‐cytokine receptor interaction”, and “ECM‐receptor interaction” (Figure 6c). Furthermore, genes involved in “ECM‐receptor interaction” and genes associated with the “TGF‐β signaling pathway” were utilized for Gene Set Enrichment Analysis (GSEA), with the gene expression pattern illustrated by a heatmap (Figure 6d–g). These data confirm that ECM‐receptor interaction and TGF‐β signaling are indeed involved in macrophages' response to lactate stimulation.

### 2.6 Identification of Potential Downstream Targets of H4K12la by Genome‐Wide CUT&Tag Analysis

**Figure 7:**
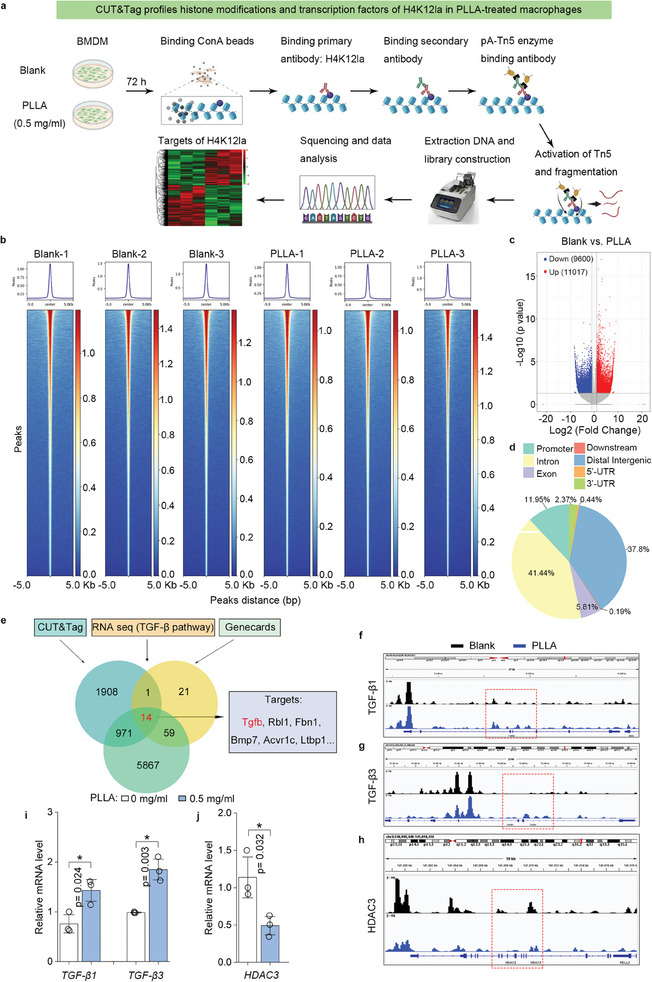
Identification of downstream targets of H4K12la by genome‐wide CUT&Tag analysis. a) Schematic of BMDMs treated with 0.5 mg mL^−1^ PLLA for 72 h and used for CUT&Tag analysis to identify the downstream targets of H4K12la. b) The binding density of H4K12la was visualized by deepTools: the heatmap presents the CUT&Tag tag counts on the different H4K12la binding peaks in BMDMs between control group and treated group. c) A volcano plot was generated to visualize the overall changes in H4K12la‐binding peaks between control BMDMs or PLLA‐treated BMDMs. d) The genome‐wide distribution of differential H4K12la‐binding peaks was analyzed between control BMDMs and PLLA‐treated BMDMs. e) Bioinformatics analysis filtered *Tgfb* as a downstream target of H4K12la in macrophages. f–h) IGV tracks for *TGF‐β1*, *TGF‐β3*, and lactylation eraser *HDAC3* from CUT&Tag analysis. i,j) qPCR assay of *TGF‐β1*, *TGF‐β3*, and *HDAC3* mRNA expression in PLLA‐treated BMDMs. ^*^ *p *< 0.05.

Histone lactylation plays a significant role in regulating the transcription of target genes.^[^ ^10^ ^]^ Genome‐wide CUT&Tag is a recently developed method for studying protein‐DNA interactions.^[^ ^22^ ^]^ Consequently, we performed CUT&Tag analysis to identify candidate genes regulated by H4K12la in macrophages. Briefly, BMDMs were treated with either 0 or 0.5 mg mL^−1^ of PLLA for 72 h. CUT&Tag analysis was carried out using antibodies against H4K12la (**Figure** **7**a), and subsequent analysis with deep tools revealed an enrichment of H4K12la peaks in macrophages (Figure 7b). The genome‐wide distribution of up‐regulated H4K12la‐binding peaks in control BMDMs and PLLA‐treated BMDMs is illustrated in Figure S2a (Supporting Information). To assess the epigenetic modulatory effects of H4K12la in macrophages in response to lactate stimulation, target genes with differential binding peaks (Figure 7c) were classified by GO and KEGG analyses. Notably, we found that the up‐regulated peak‐related genes were enriched in multiple functions and pathways associated with “binding”, “cellular component”, “protein binding”, “cellular component biogenesis”, and “Focal adhesion” (Figure S2b,c, Supporting Information). The genome‐wide distribution of differential H4K12la‐binding peaks in BMDMs is illustrated in Figure 7d, with 11.95% of these peaks located within promoter sequences. Through a combination of CUT&Tag data, RNA‐sequencing data, and gene sets from the public open access database “Genecards”. Fourteen genes, including “*Tgfb*, *Rbl1*, *Fbn1*, *Bmp*…” were identified as potential targets for H4K12la in macrophages treated with PLLA (Figure 7e). Interestingly, CUT&Tag analysis revealed an increase level of H4K12la at the promotors of *TGF‐β1* and *TGF‐β3* (Figure 7f,g), while a decrease levels of H4K12la at the promotors of *HDAC3* was observed (Figure 7h). Furthermore, a qPCR assay was conducted to confirm the mRNA levels of *TGF‐β1*, *TGF‐β3*, and *HDAC3*. The results suggest that the expression levels of *TGF‐β1* and *TGF‐β3* were significantly up‐regulated in lactate‐stimulated BMDMs, whereas *HDAC3* mRNA levels were significantly decreased (Figure 7i,j).

### 2.7 Macrophages Enhance Collagen Synthesis in Fibroblasts by Secreting TGF‐β in the Presence of Lactate

**Figure 8:**
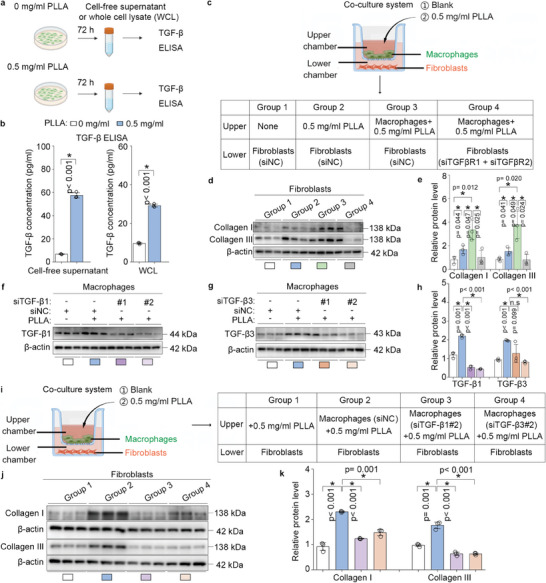
Macrophages enhance collagen synthesis in fibroblasts by secreting TGF‐β in the presence of lactate. a,b) BMDMs were treated with 0.5 mg mL^−1^ PLLA for 72 h, after which the cell‐free supernatant and whole cell lysate (WCL) were collected for TGF‐β ELISA. ^*^ *p *< 0.05. c) A flow chart of the in vitro co‐culture system, where macrophages were seeded in the upper chamber and fibroblasts in the lower chamber, TGFβR1 and TGFβR2 siRNAs were utilized to knock down the expression of TGF‐β1 receptors in fibroblasts. d,e) Western blotting analysis of collagen I and collagen III in the fibroblasts collected from the lower chamber, the relative protein levels of collagen I and collagen III were normalized to β‐actin. ^*^ *p *< 0.05. f–h) BMDMs were transfected with either TGF‐β1 siRNAs or TGF‐β3 siRNAs and subsequently treated with 0.5 mg mL^−1^ PLLA. Western blotting analysis was performed to detect the protein levels of TGF‐β1 and TGF‐β3 in BMDMs. “n.s” indicating no significance, ^*^ *p* < 0.05. i) A chart of the in vitro co‐culture system illustrates that macrophages were seeded in the upper chamber while fibroblasts were in the lower chamber, with TGF‐β1 and TGF‐β3 siRNAs used to knock down the expression of TGF‐β1 and TGF‐β3 in macrophages. j,k) Western blotting analysis of collagen I and collagen III in fibroblasts collected from the lower chamber, the relative protein levels of collagen I and collagen III were normalized to β‐actin. ^*^ *p* < 0.05.

Next, we examined whether H4K12la‐regulated TGF‐β in macrophages serves as a key effector for fibroblasts. On one hand, BMDMs were treated with 0.5 mg mL^−1^ PLLA for 72 h, and the cell‐free supernatant was collected and used for secretory proteins analysis, proteins were analyzed using Coomassie blue staining (Figure S3, Supporting Information). Additionally, 4D label‐free quantitative proteomics analysis was performed (Figure S4a, Supporting Information), revealing that three peptides of TGF‐β (ADHHATNGVVHLIDK, YLYSGQTLDTLGGK, and YLYSGQTLDTLGGKK) were identified (Figure S4b, Supporting Information). Following treatment with 0.5 mg mL^−1^ PLLA, cell‐free supernatant and whole cell lysate (WCL) from BMDMs were collected for TGF‐β ELISA. The results showed that the concentration of TGF‐β in both the cell‐free supernatant and WCL of PLLA‐treated BMDMs was significantly increased compared to the control group (**Figure** **8**a,b). On the other hand, we employed a macrophage‐fibroblast co‐culture system (Figure 8c), the expression of TGF‐β receptors, including TGFβR1 and TGFβR2, which were knocked down in fibroblasts seeded in the lower chamber using siRNA transfection (Figure S5, Supporting Information). We found that the presence of macrophages promoted the levels of collagen I and collagen III in fibroblasts following PLLA treatment. However, when the expression of TGFβR1 and TGFβR2 in fibroblasts was inhibited, these effects were reversed (Figure 8d,e). Moreover, we observed a significant increase in the protein levels of TGFβ1 and TGFβ3 in macrophages following treatment with PLLA, and we established two pairs of TGFβ1 and TGFβ3, respectively (Figure 8f–h). To further validate the impact of macrophage‐derived TGFβ1 and TGFβ3 secretion on fibroblast collagen synthesis, co‐culture experiments demonstrated that the knockdown of TGFβ1 or TGFβ3 prior to PLLA treatment resulted in a decrease in collagen synthesis in fibroblasts (Figure 8i–k). These results suggest macrophages enhance collagen synthesis in fibroblasts by secreting TGF‐β.

### 2.8 A Feedback Loop Driven by H4K12la and HDAC3 in Macrophages Activates TGF‐β Transcription and Promotes Collagen Synthesis in Fibroblasts

**Figure 9:**
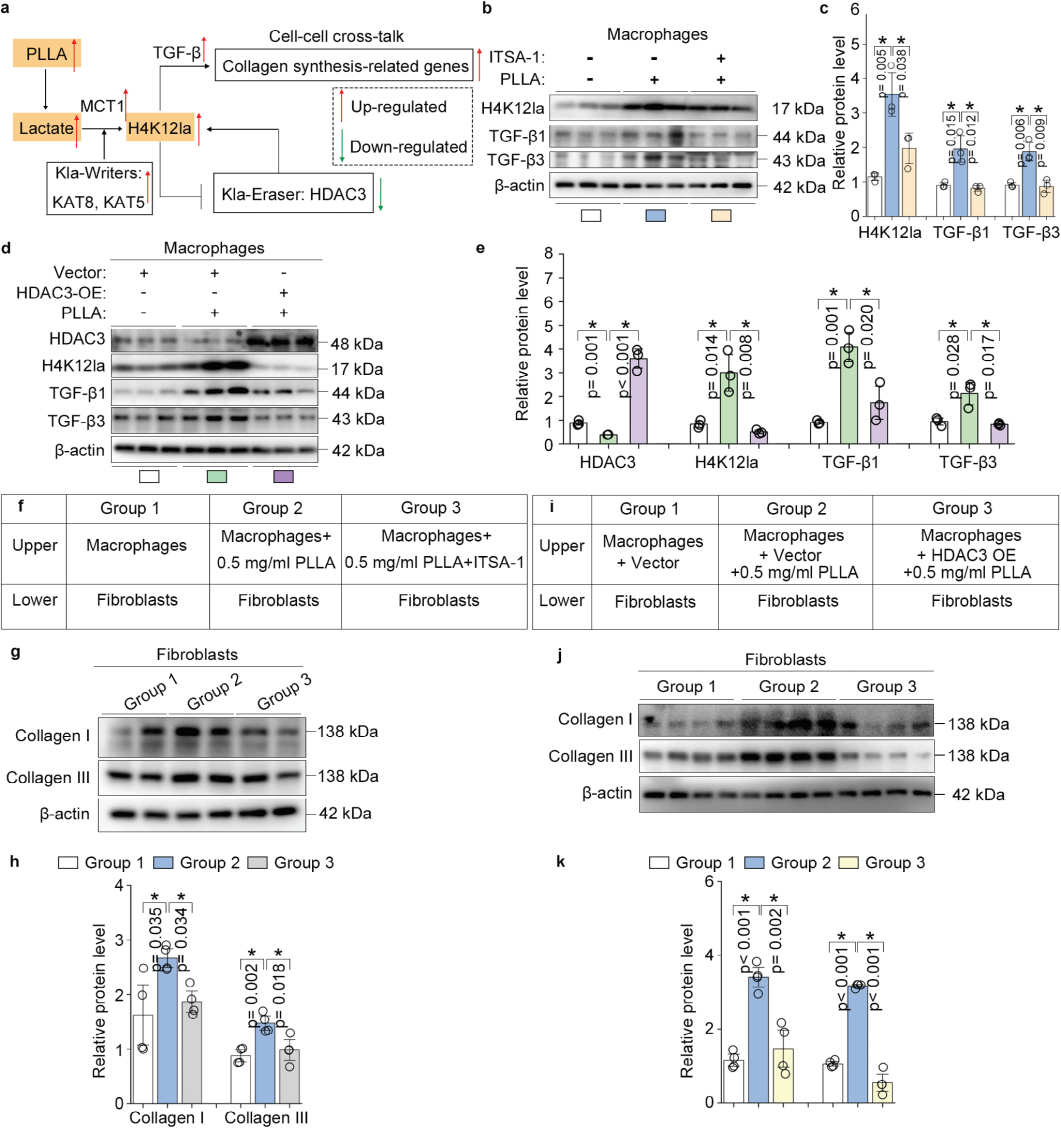
A feedback loop driven by H4K12la and HDAC3 in macrophages activates TGF‐β transcription and promotes collagen synthesis in fibroblasts. a) Schematic of the feedback loop driven by H4K12 lactylation (H4K12la) and Kla eraser HDAC3, and the regulatory relationship between H4K12la and TGF‐β. b,c) Western blotting analysis of H4K12la, TGF‐β1, and TGF‐β3 in the BMDMs treated or not treated with HDAC3 activator ITSA‐1 in the presence of PLLA, the relative protein levels were normalized to β‐actin. ^*^ *p* < 0.05. d,e) Western blotting analysis of HDAC3, H4K12la, TGF‐β1, and TGF‐β3 in the BMDMs that were either transfected with HDAC3 overexpressing plasmids or not, in the presence of PLLA, the relative protein levels were normalized to β‐actin. ^*^ *p* < 0.05. f) Using the in vitro co‐culture system, macrophages were seeded in the upper chamber, and treated or not treated with HDAC3 activator ITSA‐1 in the presence of PLLA, whereas fibroblasts were seeded in the lower chamber. g,h) Western blotting analysis of collagen I and collagen III in the fibroblasts collected from the lower chamber, the relative protein levels of collagen I and collagen III were normalized to β‐actin. ^*^ *p* < 0.05, refer to f. i) Using the in vitro co‐culture system, macrophages were seeded in the upper chamber, and transfected or not transfected with HDAC3 overexpressing plasmids in the presence of PLLA, while fibroblasts were seeded in the lower chamber. j,k) Western blotting analysis of collagen I and collagen III in the fibroblasts collected from the lower chamber, the relative protein levels of collagen I and collagen III were normalized to β‐actin. ^*^ *p* < 0.05, refer to i.

A previous study identified HDAC3 as an effective histone lactylation eraser (Kla‐Eraser).^[^ ^13^ ^]^ We hypothesized the existence of a feedback loop in which lactate stimulation increases H4K12 lactylation mediated by Kla‐writers KAT8 and KAT5, while decreased HDAC3 expression subsequently promotes H4K12 lactylation and enhances the transcription of the TGF‐β gene, which is crucial for collagen synthesis in fibroblasts (**Figure** **9**a). To confirm the functional impact of the lactate‐H4K12la‐HDAC3 feedback loop on macrophages, we activated HDAC3 in PLLA‐treated BMDMs using the selective activator ITSA‐1. Activation of HDAC3 led to a significant decrease in H4K12 lactylation, as well as TGF‐β1 and TGF‐β3 expression (Figure 9b,c). Similarly, plasmids the overexpress HADC3 enhance HDAC3 expression in macrophages in the presence of PLLA, while simultaneously reducing H4K12 lactylation, as well as the expression of TGF‐β1 and TGF‐β3 (Figure 9d,e). Furthermore, in a co‐culture system of macrophages and fibroblasts, we observed that the activation of HDAC3 through using ITSA‐1 (Figure 9f–h) or overexpression via plasmids transfection (Figure 9i–k) counteracted the macrophage‐promoted expression of collagen I and collagen III in fibroblasts in the presence of PLLA. Collectively, these results indicate that HDAC3 activation disrupts the lactate‐H4K12la‐TGF‐β axis in macrophages and suppresses lactate‐stimulated collagen synthesis in fibroblasts.

## 3 Discussion

Lactate is an important biological metabolite that plays a significant role in the processes of regeneration and repair.^[^ ^9^ ^]^ The following outlines the potential roles of lactate in tissue regeneration: 1) Energy supply: Lactate, a metabolic byproduct of glucose, can be utilized to generate energy through glycolysis, particularly when oxygen supply is limited. During tissue regeneration and repair, cells may require additional energy support, and lactate can fulfill some of these energy demands.^[^ ^23^ ^]^ 2) Signal conduction: Lactate also functions as a signaling molecule, participating in intercellular communication. Research indicates that lactate can influence cellular metabolism, proliferation, and differentiation, thereby impacting tissue regeneration and repair.^[^ ^24^ ^]^ 3) Regulating of the inflammatory response: Following tissue injury or inflammation, lactate accumulation may play a role in modulating the inflammatory response. Several studies suggest that lactate can regulate immune cell activation and influence both the intensity and duration of the inflammatory response.^[^ ^25^ ^]^ However, the precise mechanisms by which lactate contributes to collagen synthesis and its effects on skin aging remain poorly understood.

Our recent study revealed that lactate promotes collagen synthesis in fibroblasts through KAT8‐mediated lactylation of the non‐histone protein LTBP1.^[^ ^13^ ^]^ Histone lactylation, a recently identified lactate‐dependent epigenetic modification, represents a novel mechanism for regulating gene transcription via post‐translational protein modification.^[^ ^10^ ^]^ In this study, we found that lactate enhances skin collagen production through crosstalk between macrophages and fibroblasts. Mechanistically, we discovered a feedback loop driven by H4K12 lactylation and HDAC3 in macrophages that promotes collagen synthesis in fibroblasts in response to lactate stimulation. Lactate increases H4K12 lactylation levels and enhances its binding to the promoters of the TGF‐β genes. Treatment with an HDAC3 activator or HDAC3 overexpressing plasmids resulted in decreased levels of H4K12 lactylation levels in macrophages and suppressed collagen synthesis in fibroblasts. Collectively, these findings elucidate the mechanistic link between lactate stimulation, histone lactylation, and collagen production, suggesting a potential strategy for combating skin aging.

Numerous studies have demonstrated that lactate is closely associated with inflammation‐related diseases. For example, blood lactate levels significantly increase in patients with sepsis,^[^ ^26^ ^]^ and cellular senescence triggers intracellular acidification, leading to elevated lactate levels.^[^ ^14^ ^]^ These studies suggest that excess lactate may not be beneficial to cells. However, we recently reported that low concentrations of lactate can stimulate collagen synthesis in fibroblasts in the skin via the injection of Poly‐L‐lactic acid (PLLA).^[^ ^13^ ^]^ PLLA is a degradable lactate polymer that releases lactate slowly and persistently, and it is considered very safe for cells.^[^ ^12^ ^]^ Moreover, our previous study found that after 72 h of PLLA degradation, the lactate content in the cell medium was ≈0.06 µmol mL^−1^.^[^ ^13^ ^]^ Macrophages, an important type of immune cell, respond to foreign substances or inflammation and play a significant role in altering the behavior of other cells in response to environmental stimuli.^[^ ^17^ ^]^ Given that injection of PLLA is followed by macrophages infiltration,^[^ ^27^ ^]^ we hypothesized that collagen synthesis would be modulated by macrophages. In the present study, we found that PLLA treatment is safety for macrophages and does not affect their cell viability. Furthermore, the migration and phagocytosis capacity of macrophages were enhanced in a dose‐dependent manner following PLLA treatment. Clodronate liposomes are widely used for macrophage clearance; their mechanism involves interference with metabolic activity, leading to macrophage apoptosis or inactivation.^[^ ^28^ ^]^ After PLLA injection, clodronate liposomes were administered via intraperitoneal injection to clear macrophages in response to PLLA. We found that macrophage depletion reduces lactate‐stimulated collagen synthesis in the skin, a finding that was further confirmed using an in vitro co‐culture system. These results suggest that macrophages are indispensable for collagen synthesis in response to lactate stimulation. Lactate serves as a substrate for lactylation, we observed a marked increase in intracellular lactate levels in macrophages following PLLA treatment, as indicated by the lactate FiLa sensor, a powerful visualization tool developed by Professor Zhao.^[^ ^20^ ^]^ Our results demonstrate that lactate contributes to elevated levels of H4K12 lactylation, which depends on the entry of lactate into cells through monocarboxylate transporter 1 (MCT1) channels. MCT1 is a transmembrane protein that plays a crucial role in lactate transport across the cell membrane. It is primarily responsible for the transport of lactate and other monocarboxylic acids.^[^ ^23^ ^]^ MCT1 functions as a lactate/H+ co‐transporter, facilitating the export of lactate from the cell while also allowing the import of exogenous lactate, thereby playing a vital role in regulating the lactate balance both inside and outside the cell.^[^ ^29^ ^]^ The discovery of lactylation opens an intriguing possibility for a new avenue of post‐translational modification of proteins. Lactylation of lysine involves the conversion of lactic acid into lactyl‐CoA, followed by the binding of the lactyl group to the lysine side chain under the action of acyltransferase.^[^ ^10^ ^]^ p300 and KATs (such as KAT8, KAT2A, and KAT5) are two representative histone/lysine acetyltransferase (HAT/KAT) enzymes in mammalian cells.^[^ ^30^, ^31^, ^32^ ^]^ Recent reports indicate that these enzymes possess multiple acyltransferase activities, including acetylation and lactylation. Additionally, alanyl‐tRNA synthetase (AARS1) has been identified as a novel lactate sensor and functions as a lactyl‐transferase enzyme.^[^ ^15^ ^]^ Our findings demonstrate that the levels of KAT8 and KAT5 were significantly increased in PLLA‐treated macrophages. Silencing either KAT8 or KAT5 reduced the lactylation of H4K12, indicating that KAT8 and KAT5 cooperatively mediate lactyl‐CoA transfer in response to lactate stimulation.

Histone lactylation is known to regulate gene transcription. Using CUT&Tag analysis, an enzyme‐tethering strategy that provides efficient, high‐resolution sequencing libraries for profiling diverse chromatin components.^[^ ^22^ ^]^ Our data revealed that H4K12la was highly enriched in the gene promoters of TGF‐β1 and TGF‐β3 in macrophages. This is closely related to the promotion of collagen synthesis in fibroblasts. TGF‐β belongs to the transforming growth factor family and is typically expressed by fibroblasts; it is an essential factor that modulates matrix synthesis and collagen production.^[^ ^33^ ^]^ Interestingly, we found that, following lactate stimulation, TGF‐β1 and TGF‐β3 levels were significantly increased in macrophages, regulated by H4K12 lactylation. Furthermore, TGF‐β secreted from macrophages could enter fibroblasts via the TGF‐β receptor (TGFβR). This was confirmed in our study using knockdown of TGFβR expression in fibroblasts and 4D label‐free quantitative proteomics to identify proteins in the cell‐free supernatant of PLLA‐treated macrophages.

Similar to the regulatory mechanisms of other post‐translational modifications, lactylation is modulated by various regulatory enzymes, including lactyl‐transferases (often referred to as “writers”) and delactylases (termed “erasers”).^[^ ^34^ ^]^ Histone deacetylase HDAC3 has previously been identified as an effective delactylase.^[^ ^13^ ^]^ In the present study, we observed a decrease in HDAC3 expression levels in macrophages in response to lactate stimulation. Notably, CUT&Tag analysis revealed a reduction in H4K12la enrichment at the promoters of HDAC3. Consequently, we hypothesized the existence of a feedback loop between H4K12la and HDAC3. Lactylation may influence the binding of histones to the promoters of specific genes, thereby regulating their transcription.^[^ ^26^ ^]^ We found that the activation of HDAC3 using a selective activator, ITSA‐1,^[^ ^35^ ^]^ resulted in decreased H4K12 lactylation levels and suppressed the transcription of the downstream target gene TGF‐β in macrophages, which in turn inhibited collagen synthesis in fibroblasts in the presence of PLLA. Therefore, we propose that lactylation regulates H4K12 binding to the promoters of HDAC3 and modulates its expression. Our results suggest that lactate acts as a stimulator of collagen synthesis in skin fibroblasts, with the lactate‐H4K12la‐HDAC3 axis playing a pivotal role in macrophages and regulating the expression and secretion of TGF‐β.

The limitations of our study should be noted. First, the effect of fibroblasts treated with lactate on macrophages requires further investigation. Second, the use of macrophage‐specific gene knockout mice would provide greater insight into the role and mechanism of lactate in promoting skin collagen synthesis.

## 4 Conclusion

**Figure 10:**
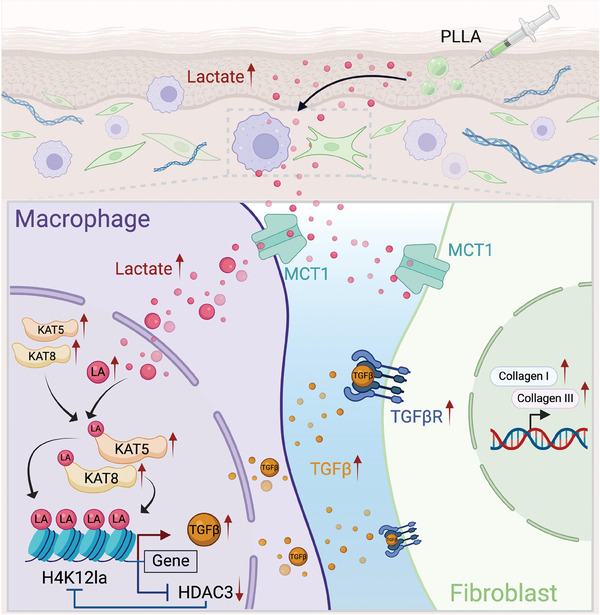
A proposed working model for how macrophage participates in lactate‐induced collagen synthesis in fibroblasts through a lactate‐H4K12la‐HDAC3‐TGF‐β axis. Graphics in Figure 10 were created using BioRender.com (https://biorender.com/).

In summary, our results demonstrate that low concentrations of lactate act as a stimulator for collagen synthesis in fibroblasts through cell‐cell crosstalk with macrophages. Following the administration of PLLA, a durable and slow‐release source of lactate, macrophages are recruited, and lactate promotes H4K12 lactylation in response to PLLA treatment. Additionally, we investigate the feedback loop between H4K12 lactylation and the lysine delactylase HDAC3 in macrophages, which regulates TGF‐β production in macrophages and collagen synthesis in fibroblasts. These findings suggest that lactate is an effective stimulator of skin collagen synthesis, and the lactate‐H4K12 lactylation‐HDAC3‐TGF‐β axis represents a novel target for anti‐skin aging interventions (**Figure** **10**).

## 5 Experimental Section

### Ethical Permission Statement

All animal use was approved by the Institutional Animal Care and Use Committee of The Affiliated Guangdong Second Provincial General Hospital of Jinan University (Approval number: 2022‐DE‐KZ‐007‐01).

### Cell Culture and Treatment

Human foreskin fibroblasts (BJ) and mouse monocyte‐macrophage cells (RAW264.7) were obtained from ATCC and cultured in high glucose DMEM (Cat#11965118, Gibco, USA), supplemented with 10% FBS (Cat#FSP500, ExCell Bio, China), 100 U mL^−1^ penicillin and 100 µg mL^−1^ streptomycin (Cat#15140122, Invitrogen, USA). Bone marrow‐derived macrophages (BMDMs) were isolated and cultured as previously described.^[^ ^36^ ^]^ Briefly, bone marrow cells were flushed with DMEM from the tibia and femur of mice aged 6–8 weeks. The cells were then cultured for 7 days in high glucose DMEM containing 10% FBS and 20 ng mL^−1^ macrophage colony stimulating factor (MCSF, Cat#RP01216, ABclonal, China) at 37 °C and 5% CO_2._ The BMDMs were identified by immunofluorescence using the macrophage marker F4/80. The RAW264.7 and BMDMs were treated with various concentrations (0, 0.25, 0.5, and 1 mg mL^−1^) of PLLA diluted in culture medium for 72 h. Additionally, 10 µm of the HDAC3 activator ITSA‐1 was employed to enhance HDAC3 expression in the presence of PLLA, while 250 nm of the MCT1 inhibitor AZD3965 was utilized to inhibit MCT1 in macrophages.

### Cell Counting Kit‐8 (CCK‐8), MTT Assay, and Calcein‐AM/PI Staining

Cell viability was assessed using the CCK‐8 assay (Cat#C0037, Beyotime, China) and MTT assay (Cat#C0009S, Beyotime, China). RAW264.7 cells or BMDMs were seeded in 96‐well plates at a density of 5000 cells per well and treated with PLLA. Following this, 10 µL of CCK‐8 reagent was added to each well, and the plates were incubated at 37 °C and with 5% CO_2_ in the dark for 2 h. The optical densities of the wells were measured using a microplate reader (Cat#Synergy HTX, BioTek, USA). BMDMs were seeded at a concentration of 5000 cells per well in a 96‐well plate. Following treatment with PLLA, 50 µL of freshly prepared MTT stock solution (5 mg mL^−1^) was added to each well, and the plate was incubated at 37 °C for an additional 4 h. After incubation, the solution was removed, and 100 µL of dissolution solution was added to dissolve the formazan crystals. The OD was then measured at 570 nm using a microplate reader. Additionally, live, and dead cell staining was performed using Calcein‐AM/PI staining. The cells were first stained with in 0.4 µm fluorescent green Calcein‐AM (Cat#BB‐4126, BestBio, China) for 30 min at 37 °C to identify live cells (green), and followed by staining with 2 µm fluorescent red PI (Cat#BB‐4126, BestBio, China) for 5 min at 37 °C to detect dead cells (red). The cells were subsequently visualized under a fluorescence microscope (Zeiss, Germany), and the number of cells was quantified in 400 µm × 400 µm fields.

### Wound Healing Assay

Cell migration was assessed using a wound healing assay. Briefly, BMDMs at a density of 2 × 10^6^/ml were cultured in a 6‐well plate and treated with either 0.25 or 0.5 mg mL^−1^ PLLA. After 24 h, the central area of the well was scratched using a sterile pipette tip to create a cell‐free zone. The scratched area was photographed using phase contrast microscopy (Zeiss, Germany) at 12 h and again at 24 h post‐scratching. The extent of scratch area was measured using Image J software (Media Cybernetics, USA).

### Phagocytosis Assay

To assess the phagocytosis capacity of macrophages stimulated with lactate, BMDMs were seeded in 24‐well plates at a density of 1× 10^5^ cells/well and treated with either 0.25 or 0.5 mg mL^−1^ PLLA. Subsequently, the cells were exposed to 0.1 mg mL^−1^ fluorescent lumispheres (1 µm diameter, Cat#7‐3‐0100, BaseLine Chromtech, China). After rinsing three times with PBS to eliminate any lumispheres attached to the cell surface, the BMDMs were fixed with 4% PFA and stained with F4/80 to delineate the out‐lines of macrophages. The number of lumispheres within each macrophage was then quantified.

### Experimental Animals and Drugs Injection Schedule

Male C57BL/6 mice (12‐months‐old) were obtained from Cyagen (Suzhou, China). PLLA (LaSynPro, SinoBiom) was dissolved in sterile saline to achieve a concentration of 50 mg mL^−1^ prior to administration. The mice were divided into four groups with three mice per group. Mice were anesthetized with 180 mg kg^−1^ of tribromoethanol (Cat#T48402, Sigma Aldrich, USA), and the back skin was shaved before the injection. A total of 100 µL of PLLA was injected into the dermal layer on the back of each mouse using a syringe, while control mice received an equal volume of saline. To deplete macrophages, 100 µL of clodronate liposomes (Cat#40337ES05/10, YEASEN, China) were injected intraperitoneally (i.p.) one day before the PLLA injection, and on days 2 and 3 after the PLLA injection, PBS was administered to the control group. At day 30, the mice were humanely euthanized under respiratory anesthesia, and skin samples were collected for subsequent experiments.

### Macrophages‐Fibroblasts Co‐Culture System

Cells were co‐cultured using a chamber (JET Biofil, China). For the co‐culture of macrophages and fibroblasts, 1 × 10^5^/ml BMDMs were cultured in the upper chamber, while 2 × 10^5^/ml fibroblasts were cultured in the lower chamber. BMDMs were either treated or untreated with PLLA, to investigate the effect of macrophages on collagen synthesis in fibroblasts in the presence of PLLA. To investigate whether TGF‐β secreted by macrophages promotes collagen synthesis in fibroblasts, macrophages in the upper chamber were transfected with either TGF‐β1 or TGF‐β3. Meanwhile, fibroblasts in the lower chamber were transfected with TGFβR1 and TGFβR2 to inhibit the receptors of TGF‐β. The sequences of the siRNAs used in this study are provided in Table S1 (Supporting Information).

### Lactate Levels Measurement and Lactate Visualization Using FiLa Sensor

Cell‐free supernatant and whole cell lysates (WCL) from PLLA‐treated BMDMs were collected after 72 h to measure lactate levels. The lactate levels were quantified using a kit (Cat#BL868A, Biosharp, China), the collected lysates were used to measure lactate levels following the manufacturer's instructions and calculating the concentrations based on a standard curve. Additionally, intracellular lactate was visualized using an ultrasensitive lactate sensor FiLa. The FiLa and FiLa‐C plasmids were generously provided by Professor Yuzheng Zhao. For virus packaging, the FiLa‐C and FiLa plasmids were transfected into HEK293T cells with psPAX2 packaging plasmid and the Pmd2.G envelope plasmid utilizing Lipofectamine 3000 (Cat#L3000015, Invitrogen, USA). After 72 h, viral supernatants were collected, concentrated, and purified via ultracentrifugation for macrophages transfection. For the visualization of intracellular lactate in BMDMs, the cells were plated in a 35 mm glass‐bottom dish, and viral supernatants were mixed with the culture medium at a 1:1 ratio, before being added to the dish. After 3 to 4 days, green fluorescence in BMDMs was observed using a fluorescence microscope. Subsequently, the cells were treated with puromycin for 48 h, followed by a change to fresh medium. Images were captured using a Zeiss confocal laser‐scanning microscope, employing a 405‐nm excitation laser and a 488‐nm excitation laser with an emission range of 500–550 nm for dual‐excitation ratio imaging.

### In Vitro siRNA and Plasmids Transfection

The silencing of MCT1, MCT14, KAT8, and KAT5 in BMDMs, as well as the silencing of TGFBR1 and TGFBR2 in human fibroblasts, was accomplished using specific siRNA reagents (Tsingke Biotechnology, China), as outlined in Table S1 (Supporting Information). The cells were transfected with siRNA using Lipofectamine RNAiMAX (Cat#13778150, Thermo Fisher, USA). HDAC3 overexpressing plasmids (pECMV‐Hdac3‐m‐FLAG) were obtained from MIAOLING BIOLOGY (Cat#P5690). These plasmids were subsequently transfected into BMDMs using Lipofectamine 3000 Reagent (Cat#L3000015, Invitrogen, USA).

### RNA‐seq, CUT&Tag, and Bioinformatic Analysis

Total RNA was extracted using Trizol. The mRNA library was prepared with an Illumina TruSeq RNA sample prep kit and sequenced on the DNBSEQ‐T7 platform (BGI, China). The criteria for identifying differentially expressed genes (DEGs) included a false discovery rate (FDR) of < 0.01, fold changes of <0.5 or > 2.0, and a P value of <0.05. CUT&Tag was performed using a CUT&Tag Assay Kit (Cat#CUT‐02, Ruoyu Biotech, China) in accordance with the manufacturer's protocol. In brief, BMDMs were plated in a 6‐well plate at a density of 1 × 10^5^ cells and treated with PLLA at concentration of 0.5 mg mL^−1^ for 72 h. Subsequently, the cells were incubated with ConA beads and rabbit anti‐H4K12la antibody (1:100) at room temperature for 2 h. Following washing, the cells were incubated with anti‐rabbit secondary antibody for 1 h at room temperature. The cells were then incubated with pA‐Tn5 transposase to obtain target DNA fragments, which were amplified by PCR using adaptor primers. The PCR products were purified with DNA magnetic beads (Cat#DB‐01, Ruoyu Biotech, China) for library construction, sequencing, and bioinformatic analysis (PERSONALBIO TECHNOLOGY CO., LTD, China). The primer pairs used for library construction are detailed in Table S2 (Supporting Information).

### 5.1 Label‐Free Quantitative Proteomics

BMDMs were treated with 0.5 mg mL^−1^ PLLA for 72 h. The cell‐free supernatant was collected by centrifugation at 10000×g for 15 min and used for 4D label‐free quantitative proteomics to identify the proteins secretion profile of macrophages following lactate stimulation. After protein extraction, the proteins were analyzed using Coomassie blue staining (Cat#P0003, Beyotime, China). Trypsin digestion, and LC–MS/MS analysis were performed by PERSONALBIO TECHNOLOGY CO., LTD, China. The resulting MS/MS data were processed using MaxQuant software.

### Real‐Time Quantitative PCR (qPCR)

Total RNA was isolated using Trizol, and cDNA was generated using the qPCR RT Kit (Cat#FSQ‐101, Toyobo, Japan), Quantitative PCR (qPCR) was subsequently performed using SYBR green (Cat#FP205, TIANGEN, Beijing, China), with an annealing temperature set at 55 °C. The expression ratio for each gene relative to *β‐actin* was calculated using the 2^−△△Ct^ formula. The primer sequences utilized in this study are presented in Table S3 (Supporting Information).

### Immunofluorescence Staining

Tissue samples were fixed with 4% PFA, dehydrated in 30% sucrose overnight, and subsequently embedded in OCT for cryo‐sectioning. Cells were also fixed with 4% PFA, following permeabilization and blocking, the samples or cells were incubated with primary antibodies (Table S4, Supporting Information) overnight at 4 °C, followed by incubation with secondary antibodies (Table S4, Supporting Information) for 2 h at room temperature.

### Masson Staining

The skin sections were rinsed three times with PBS, followed by staining with Mayer's hematoxylin for 5 min. Subsequently, they were incubated in 0.5% hydrochloric acid in 70% alcohol for 5 seconds, washed in water for 30 s, and rinsed twice in distilled water. The sections were then stained with acid ponceau for 5 min, rinsed three times in distilled water, and incubated in 1% phosphomolybdic acid aqueous for 5 min, stained with 1% aniline blue for additional 5 min and dissolved in 1% glacial acetic acid for 5 min. The sections were then dehydrated with ethanol and hyalinized with xylene. Collagen was stained blue for visualization and the staining intensity was analyzed using Image J software.

### Western Blotting

Proteins were prepared in lysis buffer containing a protease inhibitor cocktail (Cat#HY‐K0010, MCE, USA). Proteins were separated using SDS‐PAGE, specific primary antibodies and corresponding secondary antibodies used in the present study are shown in Table S4 (Supporting Information).

### Quantification and Statistical Analysis

The data are presented as mean ± SEM. Differences between groups were analyzed using a two‐sided unpaired t‐test for two groups, and one‐way analysis of variance (ANOVA) followed by Tukey's multiple comparisons test for more than two groups with Origin software. Statistical significance was set at *p* < 0.05.

### Ethic Approval Statement

All protocols of this study were approved by the Ethics Committee of The Affiliated Guangdong Second Provincial General Hospital of Jinan University.

## Conflict of Interest

The authors declare no conflict of interest.

## Supporting information



Supporting Information

Supporting Information

Supporting Information

Supporting Information

Supporting Information

## Data Availability

The data that support the findings of this study are available from the corresponding author upon reasonable request.
