# Supplementary material for: A Feedback Loop Driven by H4K12 Lactylation and HDAC3 in Macrophages Regulates Lactate‐Induced Collagen Synthesis in Fibroblasts Via the TGF‐β Signaling
Source: Adv Sci (Weinh). 2025 Feb 13;12(13):2411408. doi: 10.1002/advs.202411408 (PMC11967864; doi:10.1002/advs.202411408)
Supplement: Supplementary file 1 — Supporting Information [file ADVS-12-2411408-s005.pdf]

ADVANCED SCIENCE
Open Access

Supporting Information

for *Adv. Sci.*, DOI 10.1002/advs.202411408

A Feedback Loop Driven by H4K12 Lactylation and HDAC3 in Macrophages Regulates Lactate-Induced Collagen Synthesis in Fibroblasts Via the TGF-β Signaling

Ying Zou, Mibu Cao, Meiling Tai, Haoxian Zhou, Li Tao, Shu Wu, Kaiye Yang, Youliang Zhang, Yuanlong Ge, Hao Wang*, Shengkang Luo* and Zhenyu Ju**

Supplemental figures

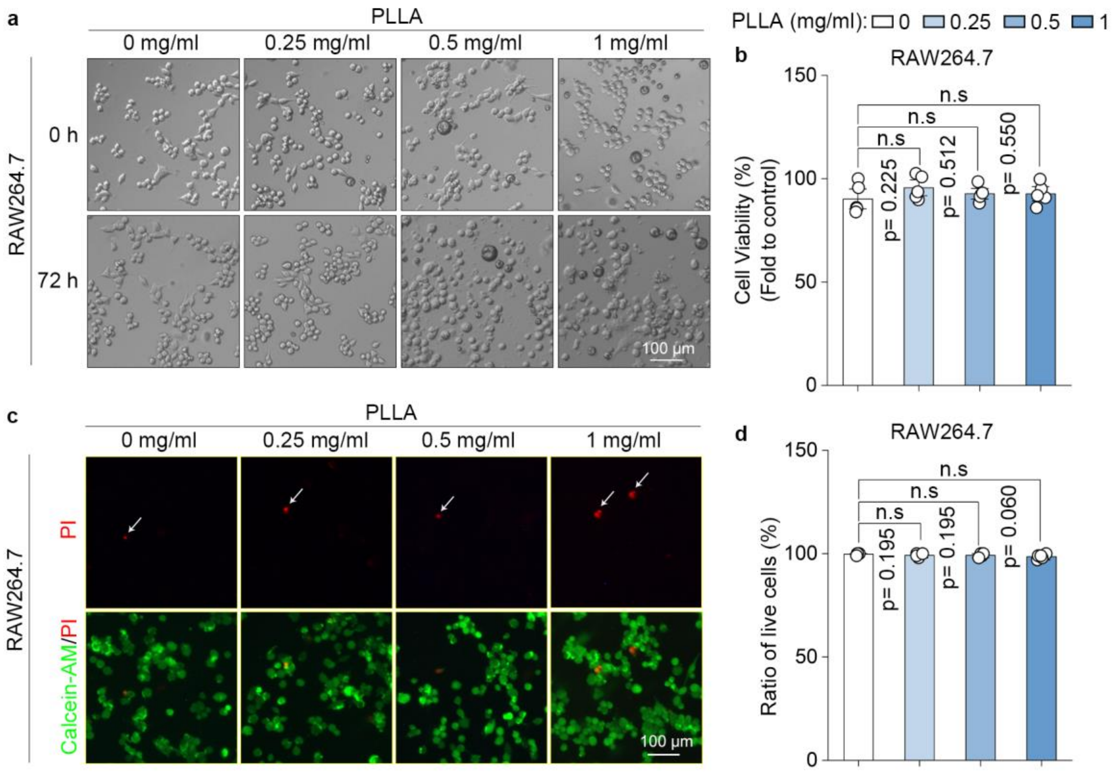

Fig.S1 (a) RAW264.7 cells were treated with varying concentrations (0, 0.25, 0.5, and 1 mg/ml) of PLLA for 72 hours. Cellular morphology was observed using a phase contrast microscope. (b) Cell viability was evaluated using the Cell Counting Kit-8 (CCK8). 'n.s' indicating no significance. (c, d) Calcein-AM/PI staining was performed to measure the percentages of live cells, 'n.s' indicating no significance.

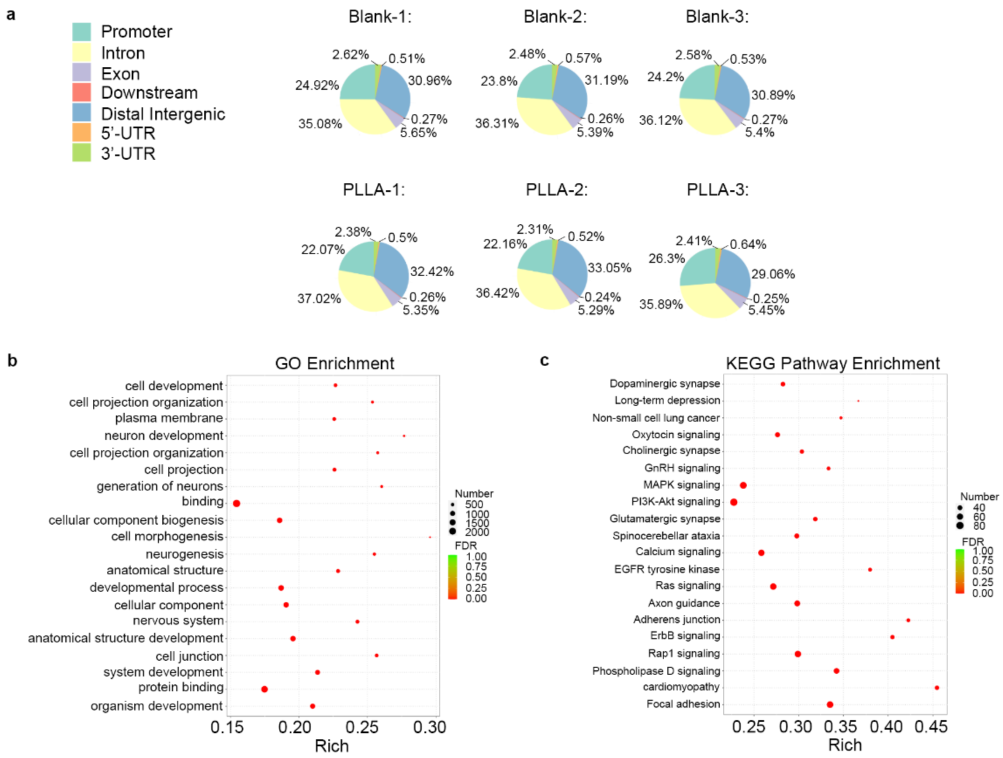

Fig.S2 (a) The genome-wide distribution of H4K12la-binding peaks in the control BMDMs and PLLA-treated BMDMs. (b) Enriched GO analysis was conducted on the up-regulated H4K12la-binding peaks. (c) Analysis of KEGG pathway enrichment was conducted on the up-regulated H4K12la-binding peaks.

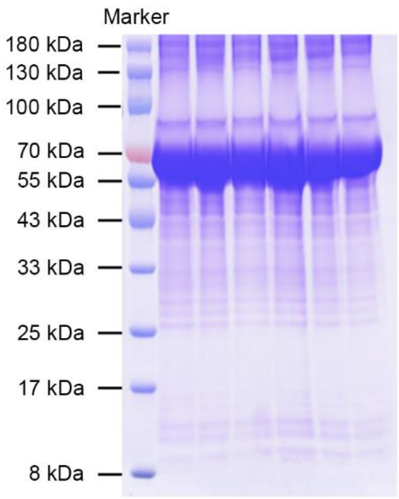

Fig.S3 BMDMs were treated with 0.5 mg/ml PLLA for 72 hours, and the cell-free supernatant was collected for secretory proteins, which were analyzed using Coomassie blue staining.

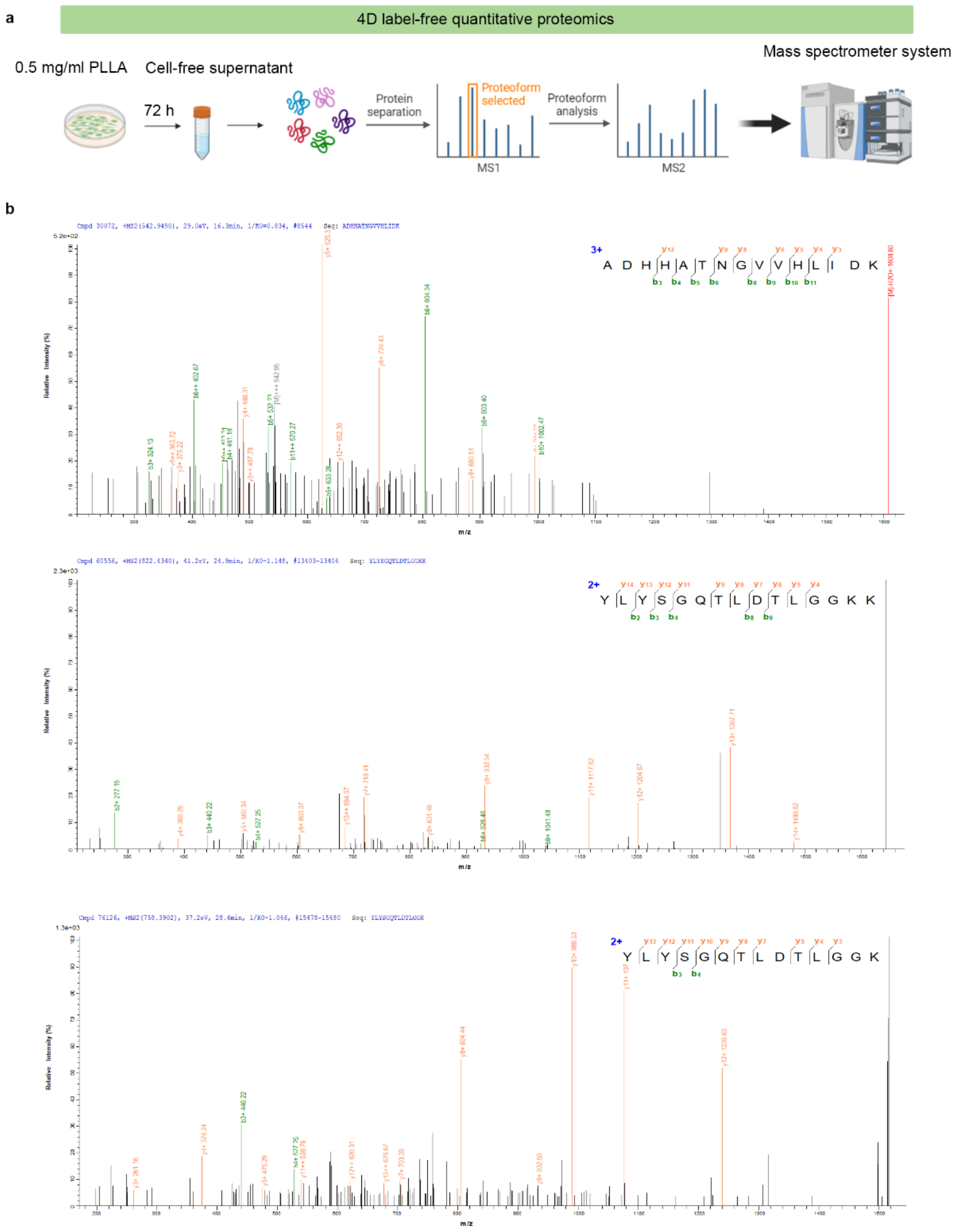

Fig.S4 (a) Schematic of BMDMs treated with 0.5 mg/ml PLLA for 72 hours, cell-free supernatant was collected and used for 4D label-free quantitative proteomics analysis to identify the secreted proteins by PLLA-treated BMDMs. (b) three peptides of TGF-β (ADHHATNGVVHLIDK, YLYSGQTLDTLGGK, YLYSGQTLDTLGGKK) were identified in the cell-free supernatant.

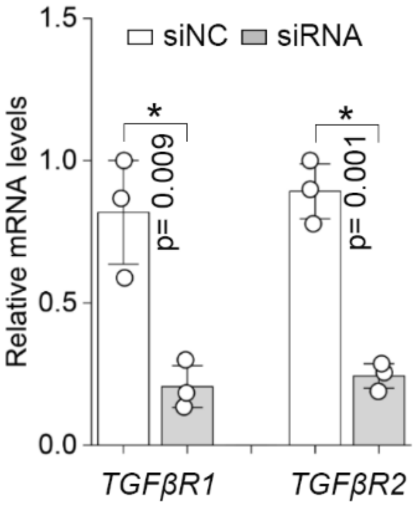

Fig.S5 qPCR analysis of *TGFβR1* and *TGFβR2* expression in siNC-transfected, TGFβR1 siRNA-transfected, TGFβR2 siRNA-transfected fibroblasts, the relative mRNA levels were normalized to β-actin. *$p < 0.05$.
