# Supplementary material for: A Feedback Loop Driven by H4K12 Lactylation and HDAC3 in Macrophages Regulates Lactate‐Induced Collagen Synthesis in Fibroblasts Via the TGF‐β Signaling
Source: Adv Sci (Weinh). 2025 Feb 13;12(13):2411408. doi: 10.1002/advs.202411408 (PMC11967864; doi:10.1002/advs.202411408)
Supplement: Supplementary file 2 — Supporting Information [file ADVS-12-2411408-s001.pdf]

ADVANCED SCIENCE
Open Access

Supporting Information

for *Adv. Sci.*, DOI 10.1002/advs.202411408

A Feedback Loop Driven by H4K12 Lactylation and HDAC3 in Macrophages Regulates Lactate-Induced Collagen Synthesis in Fibroblasts Via the TGF-β Signaling

Ying Zou, Mibu Cao, Meiling Tai, Haoxian Zhou, Li Tao, Shu Wu, Kaiye Yang, Youliang Zhang, Yuanlong Ge, Hao Wang*, Shengkang Luo* and Zhenyu Ju**

Table S1. siRNA used in this study

Gene name	siRNA sequences (5'-3')
Mouse MCT1	siMCT1#1<br>SS Sequence: GCACCGAUGUCGACGAGAAGC<br>AS Sequence: UUCUCGUCGACAUCGGUGCUG<br>siMCT1#2<br>SS Sequence: GUGUAUAUGUGUUGCUAAAUC<br>AS Sequence: UUUAGCAACACAUAUACACAU<br>siMCT1#3<br>SS Sequence: GCAGUAUCUUGGUGAAUAAAU<br>AS Sequence: UUAUUCACCAAGAUACUGCUG
Mouse MCT14	siMCT14#1<br>SS Sequence: GGUGGAUCUCUAAAUUAAAGG<br>AS Sequence: UUUAAUUUAGAGAUCCACCUG<br>siMCT14#2<br>SS Sequence: GGUCAGAGGUUCAAGGUUAGC<br>AS Sequence: UAACCUUGAACCUCUGACCCU
Mouse KAT8	siKAT8#1<br>SS Sequence: CGAAGCACAAACAAGUCAAGC<br>AS Sequence: UUGACUUGUUUGUGCUUCGG<br>siKAT8#2<br>SS Sequence: GACAGAAGUAGAUAGGCAAGG

	AS Sequence: UUGCCUAUCUACUUCUGUCAG
Mouse KAT5	siKAT5 #1:<br>SS Sequence: GGACGGAAGCGGAAAUCUAAU<br>AS Sequence: UAGAUUUCCGCUUCCGUCCAG<br>siKAT5 #2:<br>SS Sequence: GGACUUAAAGAAGAUCCAAUU<br>AS Sequence: UUGGAUCUUCUUUAAGUCCAG
Human TGFBR1	SS Sequence: AGUGCAAGUUACAAUAUUAUA<br>AS Sequence: UAAUAUUGUAACUUGCACUAG
Human TGFBR2	SS Sequence: CAAGGACAAGAACAAAGUAUG<br>AS Sequence: UACUUUGUUCUUGUCCUUGUG
Mouse TGF-β1	siTGF-β1 #1<br>SS Sequence: GAAGCGGACUACUAUGCUAAA<br>AS Sequence: UAGCAUAGUAGUCCGCUUCGG<br>siTGF-β1 #2<br>SS Sequence: GCUGCUACUGCAAGUCAGAGA<br>AS Sequence: UCUGACUUGCAGUAGCAGCGG
Mouse TGF-β3	siTGF-β3 #1<br>SS Sequence: GCGUGAACAGAGUCAUUUAGA<br>AS Sequence: UAAAUGACUCUGUUCACGCUG<br>siTGF-β3 #2<br>SS Sequence: GAAUCUUUGUAUAAAUAAAUA

	AS Sequence: UUUAUUUAUACAAAGAUUCUG
