# Supplementary material for: A Feedback Loop Driven by H4K12 Lactylation and HDAC3 in Macrophages Regulates Lactate‐Induced Collagen Synthesis in Fibroblasts Via the TGF‐β Signaling
Source: Adv Sci (Weinh). 2025 Feb 13;12(13):2411408. doi: 10.1002/advs.202411408 (PMC11967864; doi:10.1002/advs.202411408)
Supplement: Supplementary file 3 — Supporting Information [file ADVS-12-2411408-s003.pdf]

ADVANCED SCIENCE
Open Access

Supporting Information

for *Adv. Sci.*, DOI 10.1002/advs.202411408

A Feedback Loop Driven by H4K12 Lactylation and HDAC3 in Macrophages Regulates Lactate-Induced Collagen Synthesis in Fibroblasts Via the TGF-*β* Signaling

Ying Zou, Mibu Cao, Meiling Tai, Haoxian Zhou, Li Tao, Shu Wu, Kaiye Yang, Youliang Zhang, Yuanlong Ge, Hao Wang*, Shengkang Luo* and Zhenyu Ju**

Table S2. The primer pairs used for CUT&Tag library construction

Sample name	Primary antibody	Primer pairs
Blank-1	anti-rabbit IgG	N501： TAGATCGC<br>N701： TAAGGCGA
Blank-2	anti-rabbit IgG	N502： CTCTCTAT<br>N702： CGTACTAG
Blank-3	anti-rabbit IgG	N503： TATCCTCT<br>N703： AGGCAGAA
PLLA-1	anti-rabbit IgG	N504： AGAGTAGA<br>N704： TCCTGAGC
PLLA-2	anti-rabbit IgG	N505： GTAAGGAG<br>N705： GGACTCCT
PLLA-3	anti-rabbit IgG	N506： ACTGCATA<br>N706： TAGGCATG
Blank-1	anti-rabbit H4K12la	N507： AAGGAGTA<br>N707: CTCTCTAC
Blank-2	anti-rabbit H4K12la	N508: CTAAGCCT<br>N708: CAGAGAGG
Blank-3	anti-rabbit H4K12la	N509： TGGAAATC<br>N709: GCTACGCT
PLLA-1	anti-rabbit H4K12la	N510： AACATGAT<br>N710： CGAGGCTG

PLLA-2	anti-rabbit H4K12la	N511： TGATGAAA<br><br>N711: AAGAGGCA
PLLA-3	anti-rabbit H4K12la	N512： GTCGGACT<br><br>N712: GTAGAGGA
