# Supplementary material for: A Feedback Loop Driven by H4K12 Lactylation and HDAC3 in Macrophages Regulates Lactate‐Induced Collagen Synthesis in Fibroblasts Via the TGF‐β Signaling
Source: Adv Sci (Weinh). 2025 Feb 13;12(13):2411408. doi: 10.1002/advs.202411408 (PMC11967864; doi:10.1002/advs.202411408)
Supplement: Supplementary file 4 — Supporting Information [file ADVS-12-2411408-s002.pdf]

ADVANCED SCIENCE
Open Access

# Supporting Information

for *Adv. Sci.*, DOI 10.1002/advs.202411408

A Feedback Loop Driven by H4K12 Lactylation and HDAC3 in Macrophages Regulates Lactate-Induced Collagen Synthesis in Fibroblasts Via the TGF-*β* Signaling

*Ying Zou, Mibu Cao, Meiling Tai, Haoxian Zhou, Li Tao, Shu Wu, Kaiye Yang, Youliang Zhang, Yuanlong Ge*, Hao Wang*, Shengkang Luo* and Zhenyu Ju**

**Table S3. The primers used in the qPCR assay**

| Gene name | Sequences (5'-3') |
|---|---|
| Mouse TGF-β1 | Forward: CCACCTGCAAGACCATCGAC<br>Reverse: CTGGCGAGCCTTAGTTTGGAC |
| Mouse TGF-β3 | Forward: GGACTTCGGCCACATCAAGAA<br>Reverse: TAGGGGACGTGGGTCATCAC |
| Mouse HDAC3 | Forward: GCCAAGACCGTGGCGTATT<br>Reverse: GTCCAGCTCCATAGTGGAAGT |
| Human TGFBR1 | Forward: ACGGCGTTACAGTGTTTCTG<br>Reverse: GCACATACAAACGGCCTATCTC |
| Human TGFBR2 | Forward: GTAGCTCTGATGAGTGCAATGAC<br>Reverse: CAGATATGGCAACTCCCAGTG |
| Human β-actin | Forward: CATGTACGTTGCTATCCAGGC<br>Reverse: CTCCTTAATGTCACGCACGAT |
| Mouse MCT1 | Forward: TGTTAGTCGGAGCCTTCATTTC<br>Reverse: CACTGGTCGTTGCACTGAATA |
| Mouse MCT2 | Forward: GCTGGGTCGTAGTCTGTGC<br>Reverse: ATCCAAGCGATCTGACTGGAG |
| Mouse MCT3 | Forward: TAAGGCTGTGAGCGTCTTCTT<br>Reverse: GAAGCCAGAATCATGCCTGCT |
| Mouse MCT4 | Forward: TCACGGGTTTCTCCTACGC<br>Reverse: GCCAAAGCGGTTCACACAC |

| | |
|---|---|
| Mouse MCT5 | Forward: CACCTGCATCGGTGTCTTCTT<br>Reverse: AAGGAAACCACGAGGTCTCAC |
| Mouse MCT6 | Forward: ACATCTCCATAGGGGTAATCTCG<br>Reverse: TGACTACCGAACGCCTTTTGT |
| Mouse MCT7 | Forward: AGGGGTAATCTCGGGTTTAGG<br>Reverse: CCGAACGCCTTTTGTCAAAGT |
| Mouse MCT8 | Forward: CGGCTGGATAGTGGTGTTTG<br>Reverse: CAGAGTTATGGATGCCGAAGATG |
| Mouse MCT9 | Forward: TTTCAGAAGTCACCTGACGGA<br>Reverse: CAAGACCCCAACAGCTAACGG |
| Mouse MCT10 | Forward: GAGGTGGAGCTGACGAGGT<br>Reverse: CATGGACACGAAGAGCACCC |
| Mouse MCT11 | Forward: GCTTGGTCTTCTCGGCTTTC<br>Reverse: GCGAGGATGCCCCATTACC |
| Mouse MCT12 | Forward: TGCTTCCTTGTTACCATCTGC<br>Reverse: CATGCTGTTTGGGAGTAATCCT |
| Mouse MCT13 | Forward: GGGGTACTCCGTTCCTTCG<br>Reverse: GGAGGTAGCAAATGAAGCAAGC |
| Mouse MCT14 | Forward: TGAAGACGACCGAAAGGCTAA<br>Reverse: ATGTGAACAAAGAAGGACGAGAG |
| Mouse β-actin | Forward: GGCTGTATTCCCCTCCATCG<br>Reverse: CCAGTTGGTAACAATGCCATGT |
