# Supplementary material for: A Feedback Loop Driven by H4K12 Lactylation and HDAC3 in Macrophages Regulates Lactate‐Induced Collagen Synthesis in Fibroblasts Via the TGF‐β Signaling
Source: Adv Sci (Weinh). 2025 Feb 13;12(13):2411408. doi: 10.1002/advs.202411408 (PMC11967864; doi:10.1002/advs.202411408)
Supplement: Supplementary file 5 — Supporting Information [file ADVS-12-2411408-s004.pdf]

ADVANCED SCIENCE
Open Access

Supporting Information

for *Adv. Sci.*, DOI 10.1002/advs.202411408

A Feedback Loop Driven by H4K12 Lactylation and HDAC3 in Macrophages Regulates Lactate-Induced Collagen Synthesis in Fibroblasts Via the TGF-β Signaling

Ying Zou, Mibu Cao, Meiling Tai, Haoxian Zhou, Li Tao, Shu Wu, Kaiye Yang, Youliang Zhang, Yuanlong Ge, Hao Wang*, Shengkang Luo* and Zhenyu Ju**

Table S4. The antibodies used in the present study

Primary antibodies	Source	Identifier	Application
Rabbit anti-Collagen I	ABclonal	Cat#A22089	WB (1:1000)<br>IF (1:200)
Rabbit anti-Collagen III	ABclonal	Cat#A0817	WB (1:1000)
Rabbit anti-Collagen III	Proteintech	Cat#22734-1-AP	IF (1:200)
Rat anti-F4/80	Abcam	Cat#ab6640	WB (1:1000)
Rabbit anti-TGF-β1	Abcam	Cat#ab215715	WB (1:1000)
Rabbit anti-TGF-β3	ABclonal	Cat#8460	WB (1:1000)
Mouse anti-β actin	Proteintech	Cat#66009-1	WB (1:5000)
Rabbit anti-HDAC3	Biodragon	Cat#RM4568	WB (1:1000)
Rabbit anti-Pan Kla	PTM BIO	Cat#PTM-1401RM	WB (1:600)<br>IF (1:100)
Rabbit anti-H3K9la	PTM BIO	Cat#PTM-1419RM	WB (1:1000)
Rabbit anti-H3K14la	PTM BIO	Cat#PTM-1414RM	WB (1:1000)
Rabbit anti-H3K18la	ABclonal	Cat#A21214	WB (1:1000)
Rabbit anti-H3K23la	PTM BIO	Cat#PTM-1413RM	WB (1:1000)
Rabbit anti-H3K56la	PTM BIO	Cat#PTM-1421RM	WB (1:1000)
Rabbit anti-H4K8la	PTM BIO	Cat#PTM-1415RM	WB (1:1000)
Rabbit anti-H4K12la	PTM BIO	Cat#PTM-1411RM	WB (1:1000)
Rabbit anti-H4K16la	PTM BIO	Cat#PTM-1417RM	WB (1:1000)
Rabbit anti-MCT1	ABclonal	Cat#A3013	WB (1:1000)

Rabbit anti-MCT14	Proteintech	Cat#26953-1-AP	WB (1:1000)
Rabbit anti-p300	Abcam	Cat# ab275378	WB (1:1000)
Rabbit anti-KAT8	ABclonal	Cat#A3390	WB (1:1000)
Rabbit anti-KAT2A	Proteintech	Cat#66575-1	WB (1:1000)
Rabbit anti-KAT5	Proteintech	Cat#10827-1	WB (1:1000)
Rabbit anti-AARS1	ABclonal	Cat#15017	WB (1:1000)
Secondary antibodies	Source	Identifier	Application
Anti-rabbit IgG, HRP-linked Antibody	Cell Signaling Technology	Cat#7074S	WB (1:2000)
Anti-mouse IgG, HRP-linked Antibody	Cell Signaling Technology	Cat#7076	WB (1:2000)
Anti-rat IgG, HRP-linked Antibody	Proteintech	Cat#SA00001-15	WB (1:1000)
Goat anti-Rabbit IgG (H+L), Alexa Fluor™ 568	Thermo Fisher	Cat#A-11011	IF (1:400)
Goat anti-Rabbit IgG (H+L), Alexa Fluor™ 488	Thermo Fisher	Cat#A-11008	IF (1:400)
